# Schizophrenia-associated variation at *ZNF804A* correlates with altered experience-dependent dynamics of sleep slow waves and spindles in healthy young adults

**DOI:** 10.1093/sleep/zsab191

**Published:** 2021-07-30

**Authors:** Ullrich Bartsch, Laura J Corbin, Charlotte Hellmich, Michelle Taylor, Kayleigh E Easey, Claire Durant, Hugh M Marston, Nicholas J Timpson, Matthew W Jones

**Affiliations:** 1 School of Physiology, Pharmacology & Neuroscience, University of Bristol, Bristol, UK; 2 Translational Neuroscience, Eli Lilly & Co Ltd UK, Erl Wood Manor, Windlesham, UK; 3 MRC Integrative Epidemiology Unit at University of Bristol, Bristol, UK; 4 Population Health Sciences, Bristol Medical School, University of Bristol, Bristol, UK; 5 UK Centre for Tobacco and Alcohol Studies, School of Psychological Science, University of Bristol, Bristol, UK; 6 Clinical Research and Imaging Centre (CRIC), University of Bristol, Bristol, UK; 7 UK DRI Health Care & Technology at Imperial College London and the University of Surrey, Surrey Sleep Research Centre, University of Surrey, Clinical Research Building, Egerton Road, Guildford, Surrey, UK; 8 Böhringer Ingelheim Pharma GmbH & Co. KG, Biberach, Germany

**Keywords:** psychosis, schizophrenia, genetics, sleep, slow wave, spindle, motor sequence task, ALSPAC

## Abstract

The rs1344706 polymorphism in *ZNF804A* is robustly associated with schizophrenia and schizophrenia is, in turn, associated with abnormal non-rapid eye movement (NREM) sleep neurophysiology. To examine whether rs1344706 is associated with intermediate neurophysiological traits in the absence of disease, we assessed the relationship between genotype, sleep neurophysiology, and sleep-dependent memory consolidation in healthy participants. We recruited healthy adult males with no history of psychiatric disorder from the Avon Longitudinal Study of Parents and Children (ALSPAC) birth cohort. Participants were homozygous for either the schizophrenia-associated ‘A’ allele (*N* = 22) or the alternative ‘C’ allele (*N* = 18) at rs1344706. Actigraphy, polysomnography (PSG) and a motor sequence task (MST) were used to characterize daily activity patterns, sleep neurophysiology and sleep-dependent memory consolidation. Average MST learning and sleep-dependent performance improvements were similar across genotype groups, albeit more variable in the AA group. During sleep after learning, CC participants showed increased slow-wave (SW) and spindle amplitudes, plus augmented coupling of SW activity across recording electrodes. SW and spindles in those with the AA genotype were insensitive to learning, whilst SW coherence decreased following MST training. Accordingly, NREM neurophysiology robustly predicted the degree of overnight motor memory consolidation in CC carriers, but not in AA carriers. We describe evidence that rs1344706 polymorphism in *ZNF804A* is associated with changes in the coordinated neural network activity that supports offline information processing during sleep in a healthy population. These findings highlight the utility of sleep neurophysiology in mapping the impacts of schizophrenia-associated common genetic variants on neural circuit oscillations and function.

**Statement of Significance**

Convergent evidence points to abnormal sleep neurophysiology in patients with schizophrenia. To begin disentangling cause and effect, we have used a “recall-by-genotype” design to test the hypothesis that genetic variants associated with elevated schizophrenia risk also associate with altered, sleep-dependent thalamocortical network activity, even in healthy young adults devoid of psychiatric symptoms. Our findings suggest that healthy carriers of the *ZNF804A* rs1344706 risk allele harbor neurophysiological fingerprints of altered brain function that are reminiscent of the aberrant sleep EEG well-documented in patients with schizophrenia. Our study therefore lends weight to the utility of non-REM sleep as a scalable and tractable biomarker of thalamocortical circuit dysfunction in psychiatry, enabling future studies mapping routes from genetic association to brain activity and function.

## Introduction

Schizophrenia (SZ) is a debilitating psychiatric disorder with a lifetime prevalence of up to 4% [1]. SZ etiology is complex and heterogenous, but an estimated heritability of up to 80% reflects critical genetic contributions to SZ liability [2, 3]. The genetic architecture of SZ involves over 100 loci that potentially contribute to the development of the disease [4, 5]. Despite most risk variants having small individual effects and acting in combination with other genetic and environmental factors, elucidating the neuronal changes downstream of genetic liability remains crucial for understanding the etiology of psychiatric disorders.

The single nucleotide polymorphism (SNP) rs1344706 within the second intron of *ZNF804*A was the first SNP to show genome-wide significant association for psychosis in both bipolar disorder and SZ [6]. This finding has been replicated in subsequent genome wide association studies (GWAS) [4, 7–9] including a fine-mapping study which confirmed an OR for SZ of 1.10 [1.07–1.14] [10]. *ZNF804A* is expressed in the brain and predicted to encode a protein with a C2H2 zinc finger domain, indicating a role in transcriptional regulation [8, 10] and likely complex biological functions [11]. rs1344706 has been linked to several behavioral and brain phenotypes [12, 13], including altered neuroanatomy [14, 15] (but see [16] for a null result), abnormal neurophysiology [17–19] and cognition [20–22]. In particular, *ZNF804A* genotype has been associated with cortico-hippocampal functional connectivity in healthy control subjects [23, 24] and in SZ patients and their unaffected siblings [17, 25]. Therefore, though the sole contributions of *ZNF804A* polymorphisms to psychiatric risk are small, there is rationale and precedent for mapping associations between *ZNF804A* variants and brain physiology and function.

Although cognitive deficits are an established feature of SZ [26, 27], links have recently been made between cognitive symptoms and abnormal sleep. Sleep disturbances are a core feature of SZ [28, 29] and include increased sleep latency and decreased total sleep time even in untreated patients [30], hence are not caused solely by neuroleptic medication. At the neural network level, sleep in patients also features changes in electroencephalography (EEG) oscillations, particularly during NREM. Thalamo-cortical spindle oscillations are a defining feature of NREM and are reduced in patients with schizophrenia [31–34]. Consistent with the roles of spindle oscillations in memory consolidation [35–38], spindle deficits in SZ have been linked to cognitive deficits in patients [39, 40]. More recently, slow oscillations and their coordination with spindles have also been implicated in deficits in sleep-dependent memory consolidation in patients [41–43]. Again, altered NREM oscillations are evident in first-degree relatives [44], hence are not driven purely by diagnosis or medication.

Overall, there is convergent evidence that circuit abnormalities in SZ are reflected by changes in sleep physiology that, in turn, may be important for cognitive symptoms [45]. Linking specific genetic variations with sleep neurophysiology phenotypes therefore holds the promise of illuminating a broader understanding of potential mechanisms of neural circuit dysfunction in SZ. Here, we used a recall-by-genotype approach [46] to recruit healthy individuals homozygous at rs1344706, reducing issues of confounding and reverse causality common in case/control studies. We aimed to test the overarching hypothesis that, in the absence of disease, rs1344706 genotype would associate with facets of abnormal sleep neurophysiology and sleep-dependent memory consolidation seen in SZ. Previous work has (1) associated rs1344706 genotype with altered coordination of network activity during cognition [23] and (2) shown impairment of learning-dependent, coordinated slow wave (SW) activity in patients with schizophrenia [43, 47]. Our primary hypothesis therefore integrated these previous results, testing whether correlations between memory consolidation and coordinated NREM slow waves would be disrupted in the rs1344706 AA genotype group with increased genetic liability for SZ.

## Methods

The study design was published in advance [48]. Raw and processed data and metadata are available from the Avon Longitudinal Study of Parents and Children (ALSPAC) Executive Committee through a standard application process (see http://www.bristol.ac.uk/alspac/researchers/access/). Ethical approval for the study was obtained from the ALSPAC Ethics and Law Committee (ref. 9224) and The University of Bristol Faculty of Science Human Research Ethics Committee (ref. 8089). All participants provided informed consent.

### Participants

**Figure 1.**
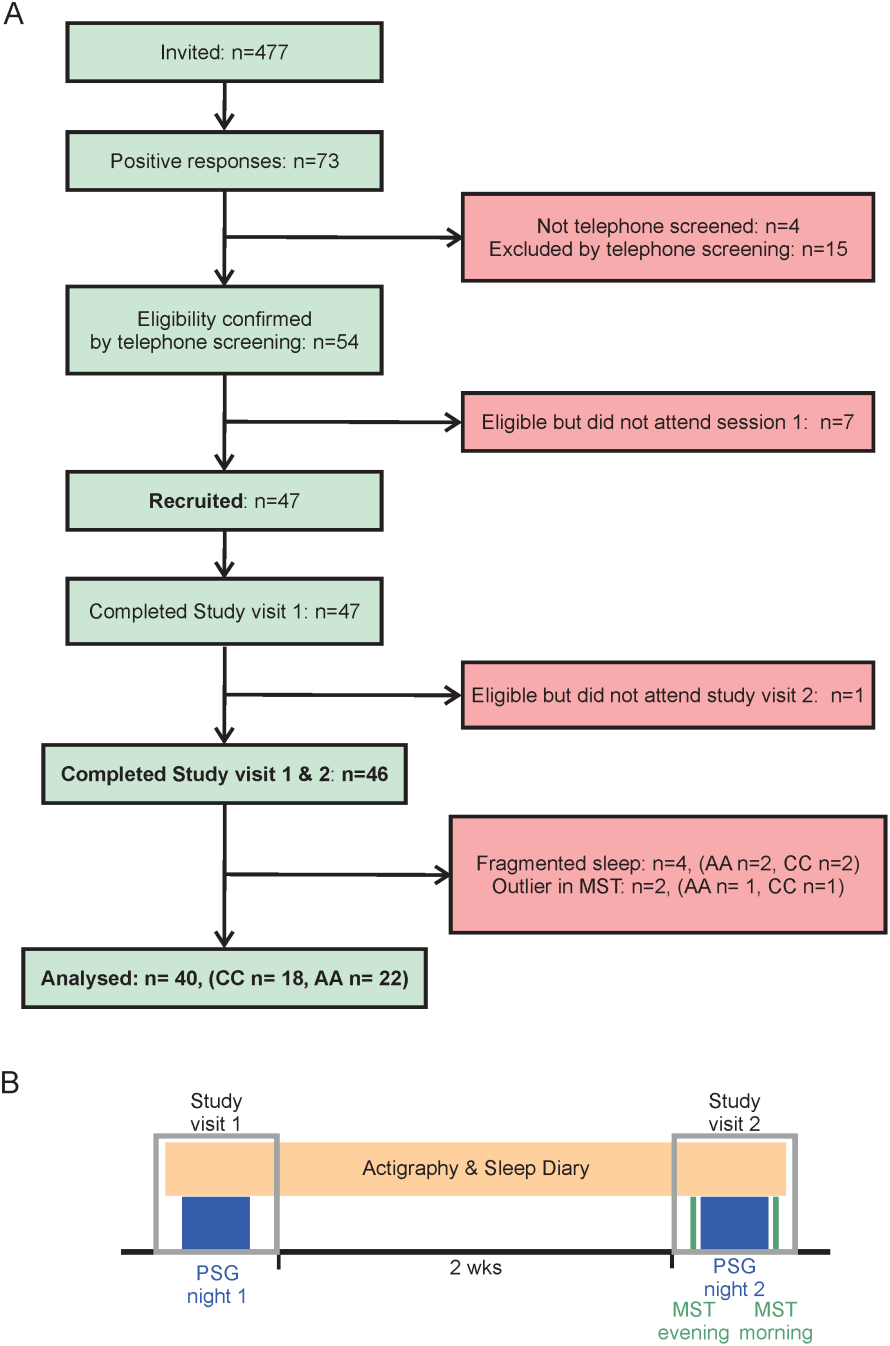
Recruitment & study design. (A) Consort flow diagram of the recruitment process: data were collected from a total of 47 participants (25 AA and 22 CC) aged 21−23 years; seven participants were subsequently excluded. (B) Study patient timeline: The study included two visits to the sleep lab at the Clinical Research & Imaging Centre in Bristol with two weeks (wks) of actigraphy monitoring between visits. Participants first visited the sleep lab for a baseline polysomnography (PSG) recording (night 1), when they were also issued an actigraphy watch to wear until the end of the second study visit. During the second visit, participants were trained on the motor sequence task (MST) in the evening and tested in the morning, with an intervening second PSG recording (night 2).

Figure 1 shows recruitment and study design. Healthy males aged 21–23 years and of European ancestry were recruited from ALSPAC, a prospective birth cohort designed to allow the study of health and development across the life course [49–51]. Pregnant women resident in Avon, UK with expected dates of delivery April 1, 1991 to December 31, 1992 were invited to take part in the ALSPAC study. The initial number of pregnancies enrolled was 14,541. Of these initial pregnancies, there was a total of 14,676 fetuses, resulting in 14,062 live births and 13,988 children who were alive at 1 year of age. When the oldest children were approximately 7 years of age, an attempt was made to bolster the initial sample with eligible cases who had failed to join the ALSPAC study originally; an additional 913 children were subsequently enrolled. The total sample size for analyses using any data collected after the age of seven is therefore 15,454 pregnancies, resulting in 15,589 fetuses. Of these 14,901 were alive at 1 year of age. The ALSPAC study website contains details of all the data that is available through a fully searchable data dictionary (http://www.bristol.ac.uk/alspac/researchers/our-data/).

Participants from the ALSPAC cohort were invited to this study based on homozygosity either for the rs1344706 allele previously associated with increased liability for SZ (AA group), or for the alternative allele (CC group). rs1344706 is located on chromosome 2 at position 185,778,428 bp (genome build GRCh37); in ALSPAC, the minor allele (C) occurs at a frequency of 40.0%. Both researchers and participants were blind to participant genotype throughout data collection.

Eligible participants were: (1) aged 20 years or over; (2) male; (3) non-smokers; (4) of European ancestry; (5) in good physical and mental health with no history of diagnosed sleep disorders; (6) able to give informed consent as judged by the investigator. Participants were excluded if: (1) they had current substance dependence (other than caffeine); (2) they had a substantive current or past illness; (3) were taking any medications that may affect or induce sleep; (4) worked at night. Participant eligibility was then verified on arrival at the sleep clinic through further standardized screening questions and completion of the Bristol Sleep Profile (BSP), and The Pittsburgh Sleep Quality Index (PSQI).

### Data collection

*Sleep lab routine and polysomnography (PSG)*. The PSG recording included nine scalp EEG electrodes placed according to the 10–20 system (at F3, Fz, F4, C3, Cz, C4, Pz, O1, and O2). Data was acquired using Cz as reference and a standard PSG recording montage using a sampling rate of 500 Hz and a high-pass filter at 0.25 Hz with an Embla N7000 amplifier and RemLogic software (Natus Medical Inc., California). Additional electrodes monitored eye movements, submental muscle activity and heart rate; video and audio were also acquired throughout the recording.

During visits, participants completed the Bristol Sleep Profile and Pittsburgh Sleep Quality Index questionnaires to assess self-rated sleep quality. Each participant also completed the Edinburgh Handedness Inventory to ascertain handedness ahead of the motor sequence task (MST). Following PSG electrode placement and bio-calibration, participants followed their usual evening routine and were encouraged to go to bed at their usual bedtime. In the morning, participants were woken as close as possible to their usual wake time. After each PSG recording, participants completed the St Mary’s Hospital Sleep Questionnaire and the Leeds Sleep Evaluation Questionnaire to assess subjective experience of their night in the sleep laboratory.

*Actigraphy*. Participants were asked to wear an ‘actiwatch’ (MotionWatch 8, CamNtech, UK) to monitor wrist movement for the entire period between their clinic visits, removing it only during water-based activities (e.g. swimming, bathing) and sports which might result in the actiwatch being damaged (e.g. rugby). Participants were also asked to keep a sleep diary.

*Motor sequence task*. On the second night, participants were trained on the MST two hours before their planned bedtime (‘training’). They were then tested again on the MST after electrode removal the following morning (‘test’). The MST is an established test of sleep-dependent memory consolidation [39, 52, 53] and was implemented in MATLAB using psychtoolbox [54], kindly donated by Dara Manoach (Harvard Medical School, Boston, MA). During the MST, participants were asked to press four numerically labeled keys on a computer keypad in a five-element sequence (4–1–3–2–4) with the fingers of their non-dominant hand, repeating “as quickly and accurately as possible” for 30 s. The numeric sequence was visible throughout the trial and dots underneath provided visual feedback for each keystroke. During both training and test sessions, participants alternated typing and resting for 30 s for a total of 12 trials. Prior to each MST session, participants completed the Stanford Sleepiness Scale to quantify vigilance levels.

### Data analyses

*Questionnaire and actigraphy data*. All paper questionnaires were manually scored and transcribed to spreadsheets. Actigraphy data were manually annotated in MotionWare (CamNtech, UK) to derive sleep architecture and circadian rhythm measures. Periods for which the participant had removed the watch were set to missing. An automated scoring algorithm determined ‘sleep onset’ and ‘sleep offset’ for each night, except where diary information and/or activity counts contradicted these times. The ‘sleep analysis’ function in MotionWare was used to derive time in bed (TIB) (total elapsed time between the ‘Lights Out’ and ‘Got Up’ times), total sleep time (TST) (the total time spent in sleep according to the epoch-by-epoch wake/sleep categorization), sleep efficiency (TST/TIB) (actual sleep time expressed as a percentage of time in bed), sleep onset latency (SOL) (the time between ‘Lights Out’ and ‘Fell Asleep’) and fragmentation index (FI) (the sum of the ‘Mobile time (%)’ and the ‘Immobile bouts ≤1 min (%)’). Measures were then averaged across all available nights for each participant.

We used non-parametric circadian rhythm analysis (NPCRA) to quantify the regularity of daily and weekly sleep wake rhythms based on inter-daily stability, intra-daily variability and amplitudes of activity [55]. A modified version of the algorithm implemented in the MotionWare software was kindly provided by Eus Van Someren (Netherlands Institute for Neuroscience), allowing periods of missing data to be excluded from the analysis. Only participants with at least seven days of data remaining after exclusions were included in the analysis. Sleep architecture and NPCRA measures derived from the actigraphy data were compared across genotype groups using a Wilcoxon rank-sum (Mann–Whitney) test.

*MST data*. The primary outcome measures from the MST were (1) the number of correct sequences (NCS) per 30-s epoch, which reflects a combination of both the speed and accuracy of performance; and (2) execution time (ET, the average time difference between successive button presses during a correct sequence) in milliseconds (ms). These measures were derived for each 30-s trial in the evening (training) and morning (test) sessions. For each outcome measure, ‘training’ performance was defined as the average of the last three training trials and ‘test’ performance was defined as the average of the first three test trials. Overnight improvement was calculated as the percentage change in each outcome measure from training performance to test performance [56].

Sleep-dependent memory consolidation was quantified using two approaches. Firstly, the mean and variance of overnight improvement measures were compared using two-sample two-sided t-tests (with unequal variances) and two-sample variance comparisons, respectively. Secondly, a linear mixed model framework was applied where training (evening) and test (morning) performance were considered repeat observations. The regression was fitted via restricted maximum likelihood (REML) using a generalized Satterthwaite approximation to estimate degrees of freedom. Session (training or test) and genotype were modelled as fixed effects, whereas participant identity was modelled as a random effect. Interactions between fixed effects were added to the final model if a likelihood ratio test comparing nested models with and without the interaction parameter suggested an improvement to model fit (p<0.05, maximum likelihood models, ML). The assumptions of the linear regression model were checked by plotting histograms and *Q*–*Q* plots of residuals from the models. In addition, a Levene’s robust test for equality of variance across groups (within session) was applied [57]. Results from the Stanford Sleepiness Scale were compared across genotype groups using a two-sample two-sided t-test (with unequal variances).

*Sleep architecture*. PSG data were manually scored by an experienced expert (blinded to participant genotype) based on AASM criteria [58] using REMLogic software (Natus Europe GmbH, Germany). Each 30 s epoch was visually classified into stages (Wake, NREM1, 2, 3, or REM). Awakenings were scored when one or more 30 s epoch was classified as wake following initial sleep onset. Individual sleep continuity and architecture was quantified using standard variables: time in bed (TIB), total sleep time (TST), sleep latency (SOL), wake after sleep onset (WASO), and sleep efficiency. Sleep stages are presented as the percentage of TST

*EEG analyses*. Prior to event detection, EEG data were-referenced to the linked mastoids and filtered with a bandpass filter in the range 0.5–30 Hz using the EEGlab function pop_eegfiltnew, which implements a zero-phase finite impulse response (FIR) filter and a Hamming window. 30s EEG epochs containing high amplitude noise and artefacts were manually removed. EEG traces were then analyzed using automatic detection of characteristic NREM sleep events—SW, delta waves, slow and fast spindle events—as described previously [43, 59]. NREM event detection relied on the same fundamental process as many other studies in the field, namely thresholding of amplitude values in a defined frequency range [32, 60, 61]. A recent version of the applied detection algorithms is freely available at: https://gitlab.com/ubartsch/sleepwalker. The extended code library for the analysis of this dataset is available upon request.

*Slow wave and delta-wave event detection*. Slow and delta waves were automatically detected after applying a 0.25–4 Hz band pass filter (using pop_eegfilt_new). The filtered EEG trace was converted to a *z*-score and all negative single wave threshold crossings with an amplitude at least 3.5 standard deviations (*SD*) above the mean amplitude were identified as candidate events. These candidate events were only accepted if they fell within the following parameter ranges: amplitude 50–300 µV; length (duration) 0.2–3 s; minimum gap between events to be considered separate was 0.5 s. Slow and delta wave events are then separated based on intrinsic frequency of the accepted candidate events (i.e. the inverse of the time difference between first peak and trough multiplied by 2): SW show an intrinsic frequency below 1.5 Hz, but delta waves intrinsic frequency is above 1.5 Hz.

**Figure 2.**
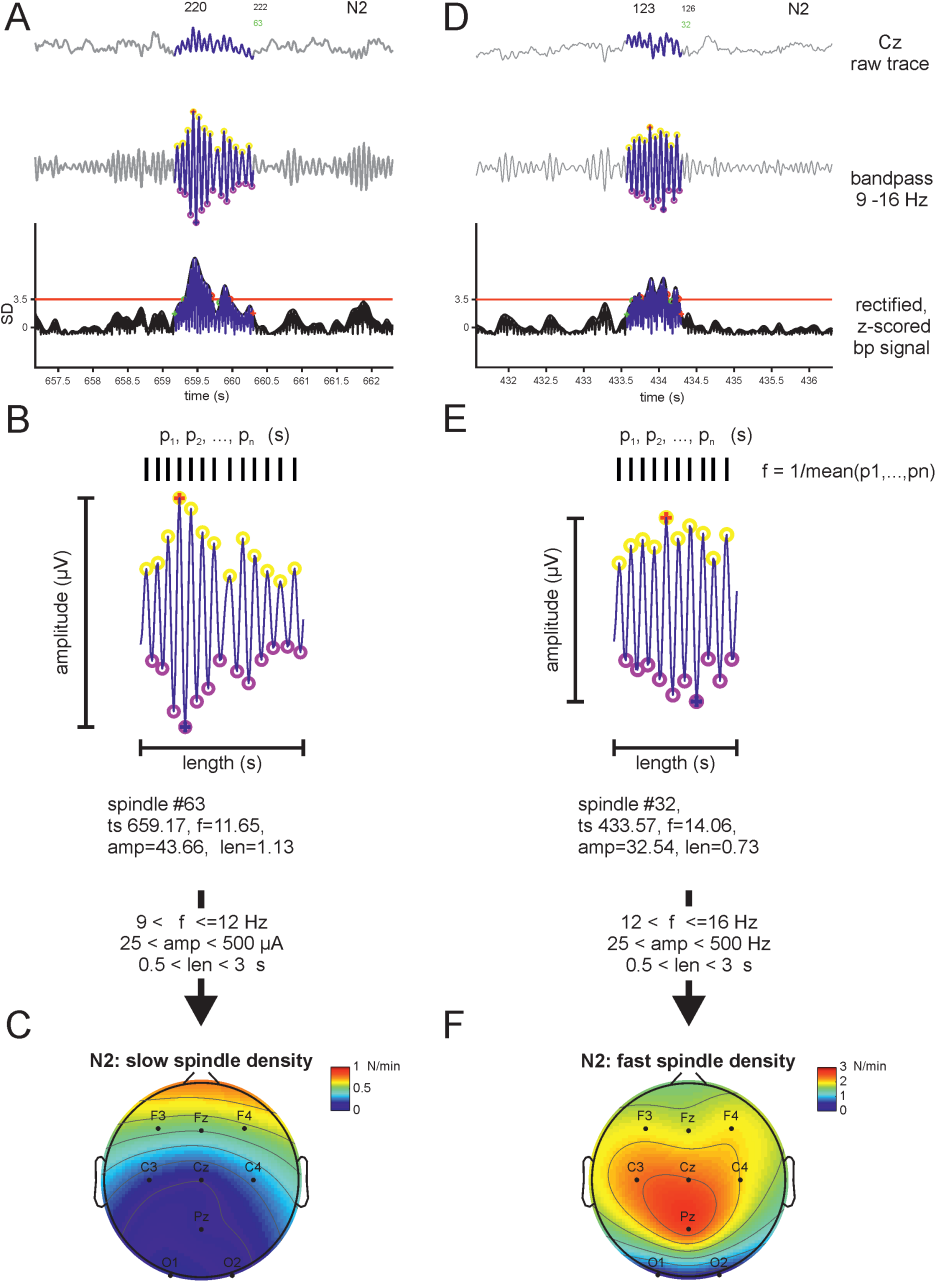
Examples of automatically detected slow and fast spindle events during NREM sleep. (A) Example of a slow spindle event, raw trace (top), bandpass filtered (middle), rectified and *z*-scored (bottom). The initial detection threshold is marked with red line at 3.5 *SD* of the whole signal (excluding noisy epochs).B) Magnification of a slow spindle event: yellow circles mark maxima, purple circles mark minima. The time difference between each peak contributes to the mean of the period, with the intrinsic frequency *f* = 1/<*p*>. Intrinsic frequency, amplitude, and duration had to meet specified selection criteria to qualify as an included spindle event. (C) Topography plot of final slow spindle density for one example participant. Note the typical frontal topography of slow spindle events. (D) Example of fast spindle event detected at the electrode Cz, format as in (A). (E) Magnification of a fast spindle event as in shown in (B). (F) Topology of all detected fast spindle events in the same participant as A−C. Note the distinct centro-parietal distribution of fast spindle events.

*Spindle detection*. Spindle events were automatically detected in sensor space EEG traces (Figure 2). The signal was filtered using a bandpass filter (9–16 Hz), the resulting filtered trace was rectified (squared), the envelope of the rectified trace was determined using spline interpolation (ML function spline) and the envelope was converted to a *z*-score. Candidate events were identified as episodes when the envelope stayed above the threshold of 3.5 for at least 50% of the estimated total event duration. Candidate events were further characterized in the time domain to determine the duration and amplitude. Spindle events were only accepted if their properties fell within the following ranges: amplitude: 25–250 µV, length (duration) 0.5–3 s.

Spindles were then classified as ‘slow’ or ‘fast’ based on their intrinsic frequency. The intrinsic frequency, *f*, of each event is determined in the time domain based on the average period between n spindle maxima *f* = 1/<*p*_(1, …, *n*)_>. Slow spindles are defined as events with 9 < *f* ≤ 12 Hz and fast spindles with 12 < *f* ≤ 16. These boundaries are based on previous studies from different groups [62–65] where the current consensus is that slow spindles exhibit an intrinsic frequency below 12 Hz.

We confirmed that spindles occurred during periods classified as sleep based on PSG, avoiding potential mis-classification of occipital alpha rhythms as spindles.

*Coherence analysis*. SW events were further characterized by applying multitaper-coherence analysis using the Chronux toolbox (www.chronux.org). For the SW triggered coherence analysis, SW negative peak times were used as *t* = 0 to collect ±2 s of raw EEG around each SW event. SW triggered coherograms were calculated using three tapers, a 1 s sliding data window, 50 ms steps and were then averaged for each electrode pair (per subject) and then averaged across genotype groups and recording night. An average SW coherence value for each electrode pair was calculated from coherograms using a [−0.5–0.5 s] and [0.5–1.5 Hz] window, and these average values were visualized as coherence matrices for each genotype group and recording night.

### Experimental design and statistics

*Experimental design*. We did not perform formal power calculations relating to our primary hypothesis about associations between genotype and slow-wave coordination, but our sample size is in line with other works associating memory with sleep EEG including, for example, work showing post-learning increases in SW coherence (based on *N* = 13, Mölle et al., 2004). Both sleep EEG and its interrelationships with behavior are age-dependent, meaning that pooling participants across a wide age range may obscure genotype-phenotype associations. The narrow age range (21–23) of ALSPAC participants in this study is therefore advantageous in this context, though findings reported here may not extend directly to other ages.

*Statistical approaches*. Tables 1 and 2 show a full record of statistical methods and their alignment to analytical arguments. Results presented are mean ± standard error (SE) unless stated otherwise.

**Table 1.** Overview of statistical analysis of non-EEG data

Characteristic	Aim	Data source	Statistical approach	Results
Confounding factors	To compare possible confounding factors across genotype groups and between recruited and invited groups	Existing ALSPAC data	Categorical variables—Pearson chi-square; continuous variables—Wilcoxon rank-sum (Mann–Whitney)	No differences (not shown)
Motor sequence task performance	To compare overnight improvement in the MST across genotype groups	MST—overnight improvement in percent	Difference in means: two-sample two-sided *t*-test (unequal variances); difference in variances: two-sample variance comparison	Figure 3 Table 3
Motor sequence task performance	To estimate the effect of genotype and session (training versus test) on MST performance	Average number of correct sequences and reaction times for last three trials (training, evening) and first three trials (test, morning).	Difference in means: linear mixed model with MST performance as the dependent variable, genotype group and session as fixed effects and individual as a random effect, difference in variances: Levene’s robust test for equality of variance	Figure 3 Table 4
Diurnal sleep wake rhythms	To compare sleep behavior and daily rhythm across genotype groups	Actigraphy	Difference in means: Wilcoxon rank-sum (Mann–Whitney)	Supplementary Table S1
Sleep architecture	To compare objectively measured sleep	PSG	Difference in means: linear mixed model with sleep architecture variables as dependent variables	Supplementary Table S2
Subjective and objective sleep quality	To compare objectively measured sleep between those who self-reported good vs. poor sleep quality	Pittsburgh Sleep Quality Index, actigraphy, PSG	Difference in means: Wilcoxon rank-sum (Mann–Whitney)	No differences (not shown)

ALSPAC, Avon Longitudinal Study of Parents and Children; MST, motor sequence task; PSG, polysomnography.

**Table 2.** Overview of statistical analysis of sleep EEG data

Characteristic	Aim	Data source	Statistical approach	Results
Spectral power of whole NREM epoch	To estimate the effect of genotype and night (first vs. second) on spectral power and to explore possible interactions between the two factors	Noise free whole epochs of N2 & N3 sleep	Linear mixed model fitted using a stepwise reduction procedure, followed by predicted marginal means analysis	Not shown
Event properties for: slow waves, delta waves, slow spindles, fast spindles	To estimate the effect of genotype and night (first vs. second) on event properties and to explore possible interactions between the two factors	NREM event properties derived from automatic detection during N2 & N3 sleep	Linear mixed model fitted using a stepwise reduction procedure, followed by predicted marginal means analysis	Figure 4, Supplementary Tables S3 & S4
				Supplementary Tables S5 & S6
				Figure 5, Supplementary Tables S7 & S8
				Supplementary Tables S9 & S10
SW event triggered slow coherence	To estimate the effect of genotype and night (first vs second) on SW event triggered SW coherence and to explore possible interactions between the two factors	SW event triggered EEG data window (± 2 s) for coherence analysis (N2 & N3)	Linear mixed model fitted using a stepwise reduction procedure, followed by predicted marginal means analysis	Figure 7, Supplementary Tables S11 & S12
Spectral coherence at SW frequency (0.5–1.5 Hz) of whole NREM epochs	To estimate the effect of genotype and night (first vs. second) on NREM coherence and to explore possible interactions between the two factors	Noise free whole epochs of N2 & N3 sleep	Linear mixed model fitted using a stepwise reduction procedure, followed by predicted marginal means analysis.	Not shown
Relation between NREM sleep properties and behavioral change	To establish predictability of overnight behavioral change from NREM sleep features	NREM event properties, SW coherence	Principal component analysis + multilinear regression	Figure 6, Table 5 Figure 8, Table 6

Our main method of statistical analysis is the linear mixed model (LMM) using maximum likelihood estimation. LMM are particularly suited for the application to repeated measurement studies with multilevel data as they are (1) tolerant to missing data, (2) allow inclusion of all collected data without averaging (which results in a loss of statistical power), and (3) allow the explicit modelling of random effects which leads to more robust model parameter estimation [66].

Behavioral measures were analyzed either by a comparison of means across groups (two-sample two-sided *t*-test or Wilcoxon rank-sum test) or by fitting a LMM with genotype and MST session (training versus testing) fitted as fixed effects, participant identity was fitted as a random effect (using Stata v14.2 [67]). The presence of interactions between fixed effects was evaluated via a likelihood ratio test comparing nested models with and without the interaction parameter.

PSG-derived sleep architecture and EEG measures were analyzed using LMM with genotype and recording night (night 1: baseline, night 2: learning) fitted as fixed effects. PSG-derived event properties or coherence measures were compared across genotype groups, electrodes/electrode pairs, recording nights (night 1: baseline, night 2: learning) and sleep stages (N2, N3) using a linear mixed model framework and a stepwise reduction procedure implemented using the *lme4* [68] and *lmerTest* [69] packages in R. We built full models of the general form [y ~ genotype + night + electrode + sleep_stage + (genotype * night) + (1|ID)], where *y* is any derived sleep variable, and then applied backward elimination of non-significant model terms using the R function *step*, which is part of the R package *lmerTest* [69]. Here we focused on night and genotype effects and their interactions, although data from all electrodes and sleep stages contributed to the final model result.

Thus, while all EEG channels were included in all statistical models, where exemplar channels are reported in the figures, this is because they represent sites of maximal SW (F3) or fast spindle (Cz) activity.

The covariance of NREM event features was estimated using Pearson’s correlation coefficient. We calculated correlation between the average of NREM event features from all electrodes for each individual and then averaged over each genotype group for recording night 2. To compare the structure of covariance matrices between genotype groups, we employed Box’s *M* test, also known as Box’s test for equivalence of covariance matrices. The test compares the covariance of two matrices of predictor variables [70]. We used an open-source implementation available at mathworks.com [71].

We employed a combination of principal component analysis (PCA) (MATLAB function *pca*) and stepwise multilinear regression (MATLAB function *stepwiselm*) to assess the relationship between NREM neurophysiology features and overnight memory consolidation. As expected, the majority of NREM neurophysiology features were highly correlated with one another (both event properties and SW coherence values). We applied PCA to reduce dimensionality and decorrelate predictor variable sets. The resulting principal components were fed into a stepwise procedure to build multilinear regression models. Model terms were added based on an F-test criterion (*p* < 0.01) and final model terms were reported.

## Results

Data were initially collected from 47 participants (25 AA and 22 CC). The two genotype groups did not differ in maternal education, social class, psychosis-like symptoms at age 18, or in the Wechsler Abbreviated Scale of Intelligence at ages 8 or 15 (not shown). Data from seven participants were excluded: (1) one participant did not attend the second clinic visit; (2) four participants showed highly fragmented sleep during at least one recording night as identified by expert reviewing of video PSG data (high WASO and arousals; 2 AA and 2 CC); (3) two participants were outliers based on performance in the MST (>*Q*3 + 1.72*IQR or <*Q*1 – 1.72*IQR, 1 AA and 1 CC). We therefore present results for 40 participants.

### Increased variability in motor sequence task consolidation in the AA carriers

**Figure 3.**
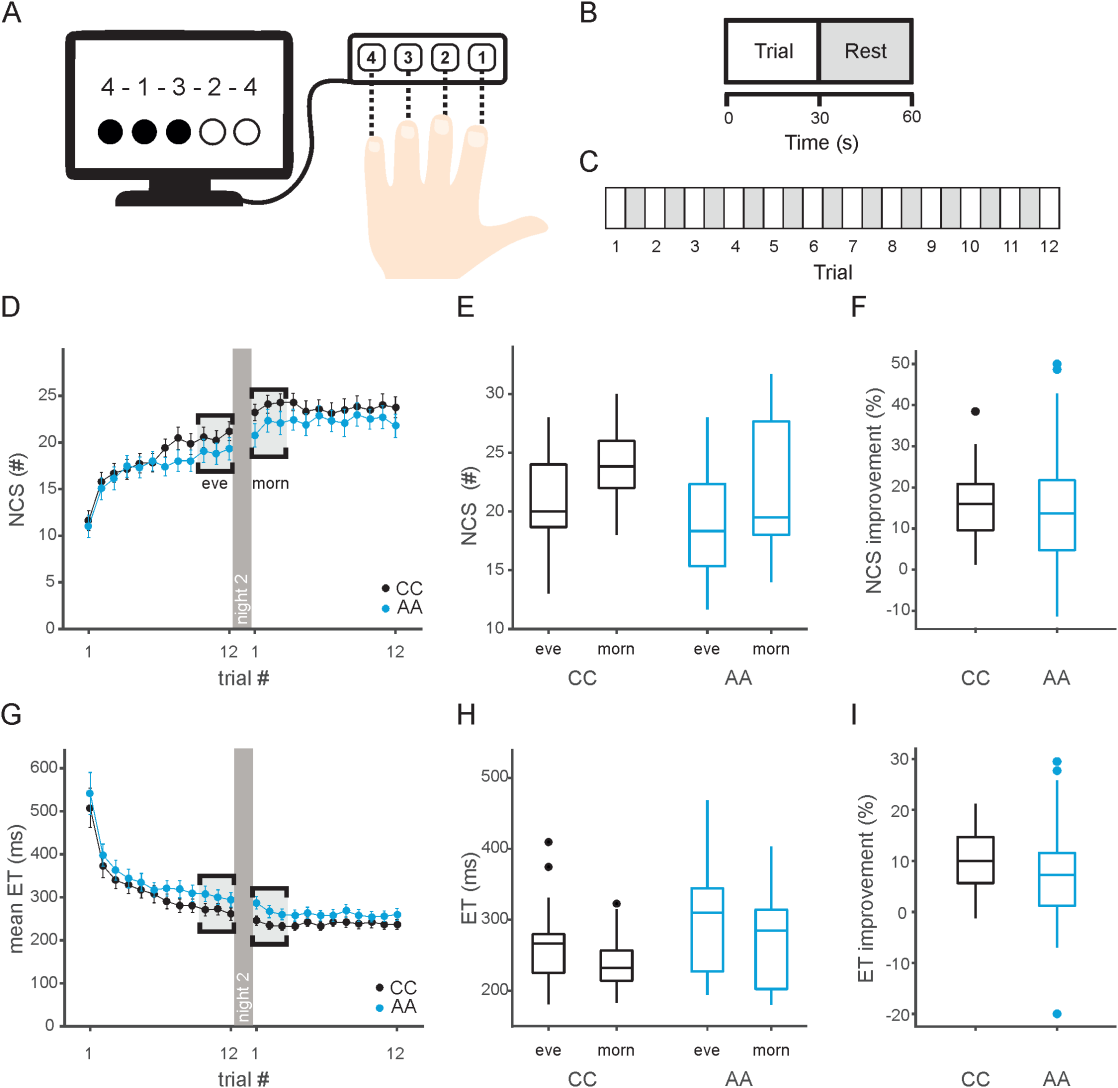
Sleep dependent consolidation of motor sequence learning. Black: CC group (*N* = 18); Blue: AA group (*N* = 22). (A) Motor sequence task (MST) experimental setup: Participants were asked to type the sequence 4−1−3−2−4 as quickly and accurately as possible on a modified computer number keypad. (B) Each trial lasts 30 s, trials are interspersed with 30-s rest periods. (C) Each participant has a total of 5 min and 30 s response time to complete 12 trials in total. (D) MST learning curves showing the number of correct sequences per trial. Night 2 is indicated by a dark grey separator, the last three and first three trials used to calculate the average for the evening (eve) and morning (morn) performance are highlighted in grey. (E) Box plot showing median number of correct sequences (last three trials in the evening vs. first three trials in the morning) for each MST session and genotype group. (Plots indicate the median, with boxes showing the 25th and 75th percentile of data, whiskers indicate the range of values inside 1.5 × outlier range, extreme values (outside 1.5 IQR) are plotted as individual data points). (F) Boxplot showing the median of overnight improvement in number of correct sequences/30 s trial as percentage change from evening to morning performance. (G) Learning curves as in A but for the mean execution time (ET, button press latency within a correct sequence) per trial. H) Boxplot showing the median ET during correct sequence button presses (last three trials in the evening vs. first three trials in the morning) for each MST session and genotype group. (I) Boxplot of median overnight improvement in ET measured as absolute percentage change from evening to morning performance

On the second study visit (night 2), participants were asked to complete the MST in the evening before going to bed, and again in the morning after waking up (Figure 3, A–C). Participants did not differ in the Stanford Sleepiness Score when performing the task (Table 3).

**Table 3.** Stanford sleepiness score and MST improvement

	CC group (*N* = 18) Mean (*SD*)	AA group (N=22) Mean (*SD*)	*t*-test *p*^b^	Levene’s test *p*^c^
**Stanford Sleepiness Score**				
Evening	2.5 (0.9)	3.1 (1.4)	0.11	0.05
Morning	2.9 (1.0)	2.5 (0.9)	0.22	0.61
**MST improvement** ^a^				
NCS (#)	16.9 (9.6)	15.9 (16.8)	0.82	0.02
ET (ms)	–10.5 (6.2)	–8.3 (11.9)	0.45	0.01

NCS = mean number of correct sequences, ET = mean execution time during correct sequences.

^a^ Improvement calculated as the percentage change in each outcome measure from training performance to test performance.

^b^ *p*-value from two-sample two-sided t-test (unequal variances).

^c^ *p*-value from Levene’s robust test for equality of variance across groups, (Levene, 1960).

Overall performance levels for practice-dependent increases in the number of correct sequences (NCS)—and corresponding decreases in button press latency within correct sequences (‘execution time’, ET) —were comparable between genotype groups. Figure 3, D and G show the MST learning curves for both genotype groups and Figure 3, E and H show the averages of the last three trials in the evening and first three trials in the morning that are used to calculate overnight improvement.

Participants in both groups improved overnight as quantified by the mean NCS (overnight change in NCS, CC: 16.9 ± 9.6%, AA: 15.9 ± 16.8%, Figure 3, F, Table 3, mean ± *SD*) and mean ET (overnight change in ET, CC: 10.5% ± 6.2%, AA: 8.3% ± 11.9%, Figure 3, I, Table 3, mean ± *SD*).

Linear mixed modelling of the MST performance data confirmed effects of session (training vs. test) on NCS (session: *F*(1, 39)= 79.1, *p* = 6.38e−11) and ET (session: *F*(1, 39) = 28.8, *p* = 3.93e−06), suggesting sleep-dependent consolidation of motor memory in both genotype groups. There was no strong evidence for an effect of genotype on task performance, but point estimates suggested that AA group participants produced fewer correct sequences (*F*(1, 38) = 1.61, *p* = 0.21) and had slower execution times (*F*(1, 38)= 3.0, *p* = 0.09, Table 4).

**Table 4.** Linear mixed model analysis of MST performance across groups and sessions

	Mean (*SD*)				Levene’s test		Linear mixed model	
	CC group (*N* = 18)		AA group (*N* = 22)		*p* ^a^		Session	Genotype
	Train	Test	Train	Test	Train	Test	Beta^b^ (SE), *F*(df1, df2), *p*	Beta^c^ (SE), *F*(df1, df2), *p*
NCS (#)	20.7 (4.1)	23.9 (3.6)	19.1 (5.3)	21.7 (5.5)	0.16	0.02	*β* = 2.91 (0.33), *F* = 79.1 (1, 39), *p* = 6.38 x 10^-11^	*β* = –1.87 (1.48), *F* = 1.61 (1, 38), *p* = 0.21
ET (ms)	268 (58)	238 (41)	301 (80)	271 (62)	0.07	0.02	*β* = –30 (6), *F* = 28.8 (1, 39), *p* = 3.93 × 10^-06^	*β* = 33 (19), *F* = 3.0 (1, 38), *p* = 0.09

NCS = mean number of correct sequences, ET = mean execution time during correct sequences.

^a^ *p*-value from Levene’s robust test for equality of variance across groups (within session).

^b^ Given with respect to the training session (evening) performance as baseline.

^c^ Given with respect to the CC group as baseline.

The AA group showed higher variance in overnight improvement in NCS (SD CC: 9.6, AA: 16.8, two-sample variance comparison *p* = 0.02, Table 3) and ET (SD CC: 6.2, AA: 11.9, two-sample variance comparison *p* = 0.01). This higher variance was particularly pronounced during the morning test session (SD NCS, CC: 3.6, AA: 5.5, Levene’s test *p* = 0.02; SD ET, CC: 41 ms, AA: 62 ms, Levene’s test *p* = 0.02).

### Sleep timing, architecture, and quality appear unaffected by rs1344706 genotype

We did not find evidence for consistent effects of genotype on diurnal rhythmicity derived from actigraphy (Supplementary Table S1) or on subjective sleep quality derived from questionnaires (not shown). PSG-derived measures of sleep architecture and quality also appear unaffected by rs1344706 genotype, with mean sleep efficiency ranging from 90% to 93% for both nights and groups (Supplementary Table S2).

### Slow wave amplitudes depend on experience, but only in the non-risk CC group

**Figure 4.**
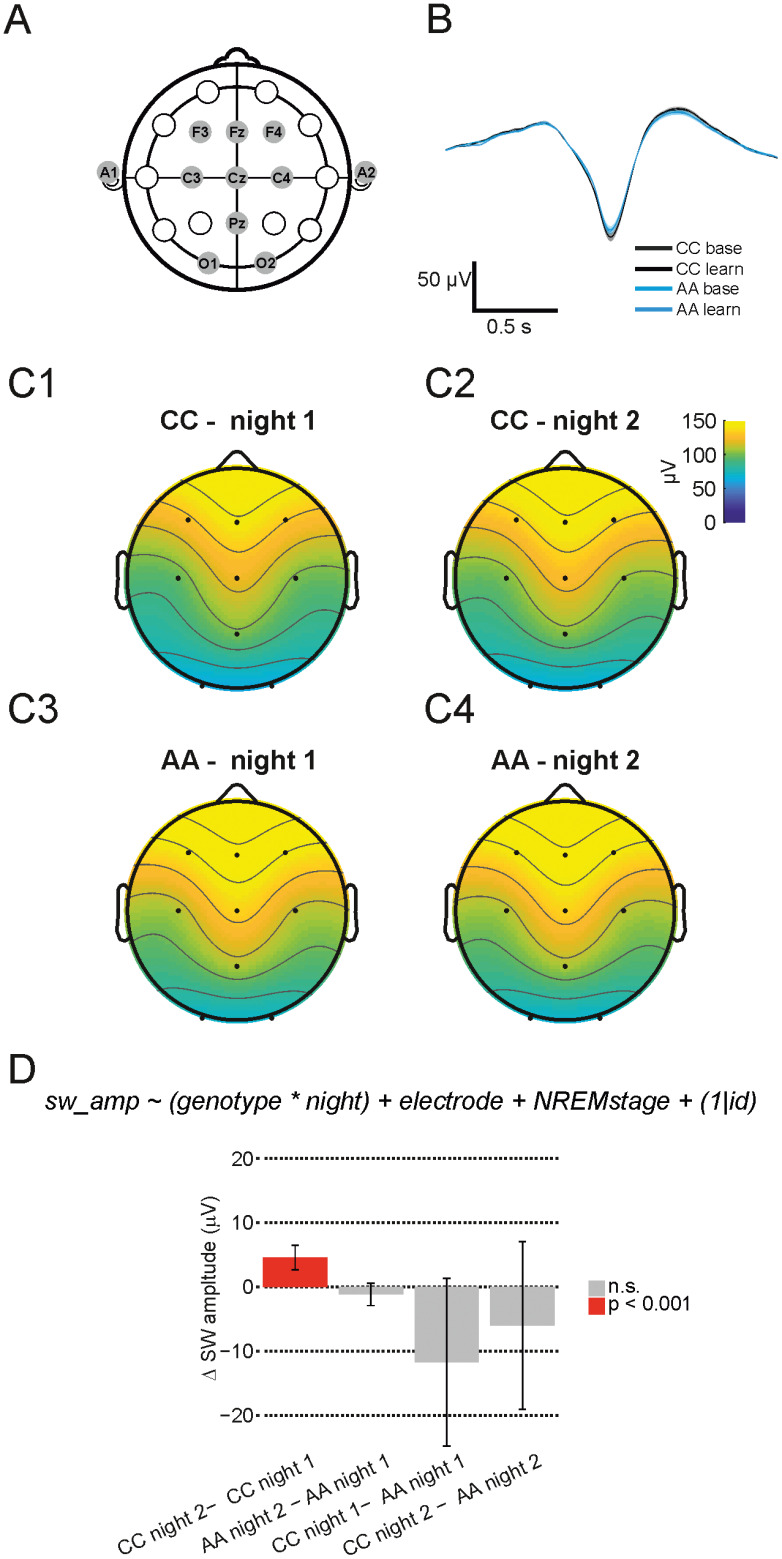
Slow wave events increase in amplitude after learning only in CC carriers. (A) EEG electrodes were placed in standard locations according to the 10−20 system. (B) SW wave-triggered average at electrode F3 during N3 sleep for both genotype groups and recording nights. CC, baseline night, grey, CC, learning night, black, AA baseline night, light blue, AA learning night, dark blue. We found no difference for any time bin (*p* < 0.05, Wilcoxon rank sum test, no correction). (C1−4) Topography plots of SW amplitudes at all recorded EEG electrodes for both genotype groups and recording nights. (D) Estimated marginal means differences for the factors genotype and night derived from a linear mixed model analysis of all detected SW amplitudes (see Methods for details). The CC group show an increase in SW amplitude during night 2, but AA do not. Error bars indicate 95% confidence intervals.

To assess potential neurophysiological correlates of variance in MST performance, we used custom detection algorithms to extract SW (0.5−1.5 Hz) events in EEG traces recorded during whole night polysomnography (Figure 4, A). These exploratory analyses are secondary to testing our primary hypothesis regarding SW coordination, but aid interpretation of the coherence results in Figures 7 and 8. Figure 4, B shows averaged, SW triggered, average EEG traces for both genotype groups and nights at electrode Fz during N3 sleep, indicating SW morphology was comparable between genotypes groups.

**Figure 5.**
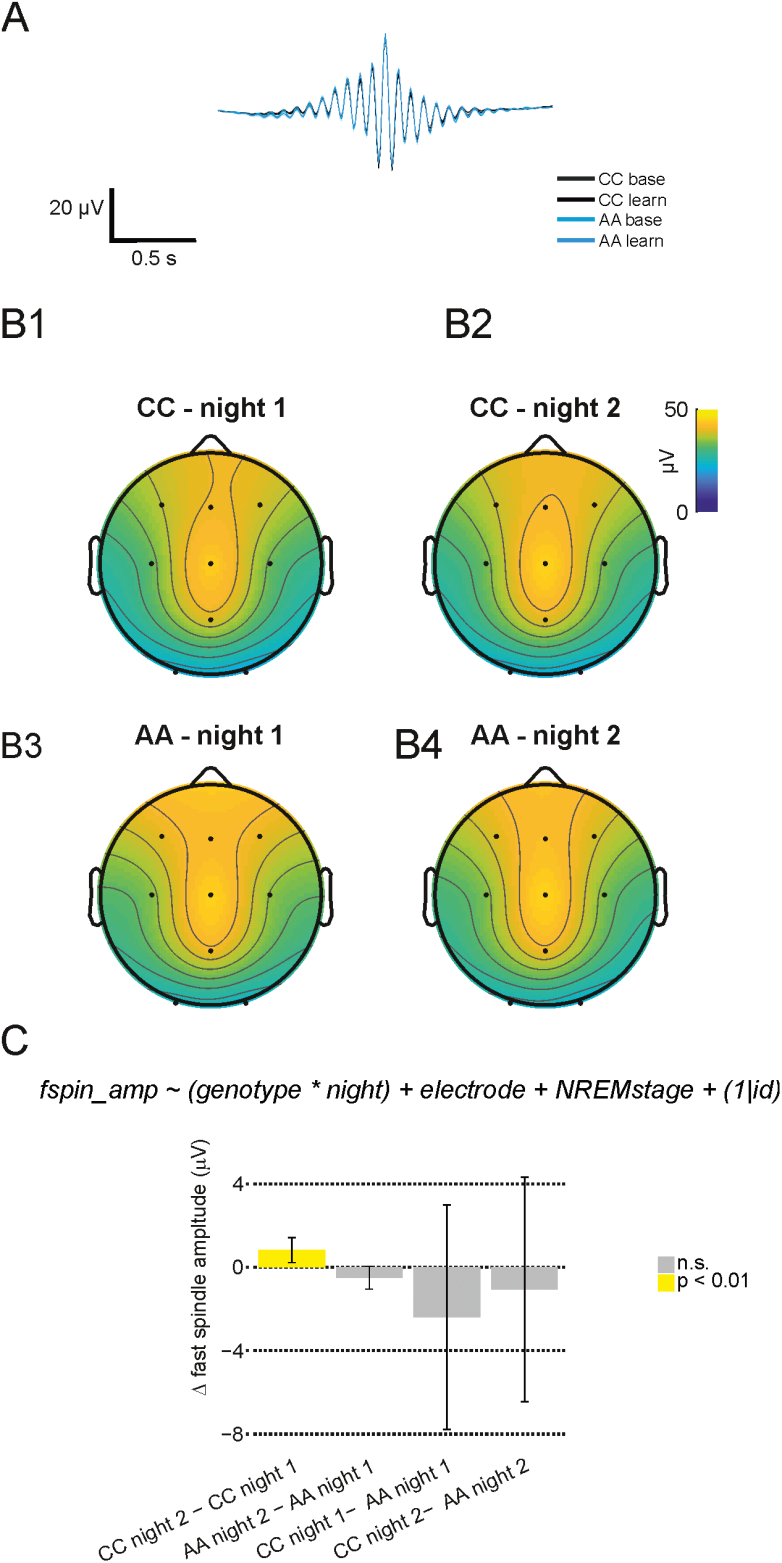
Fast spindle events increase in amplitude after learning only in CC carriers. (A) Spindle wave-triggered average at Cz for all genotype groups and both recording nights. We found no difference for any time bin (*p* < 0.05, Wilcoxon rank sum test, no correction). (B) Average spindle amplitude topography plots for both genotype groups and recording nights. (C) CC individuals show an increase in fast spindle amplitude in night 2, but AA’s do not. Error bars indicate 95% confidence intervals.

**Figure 6.**
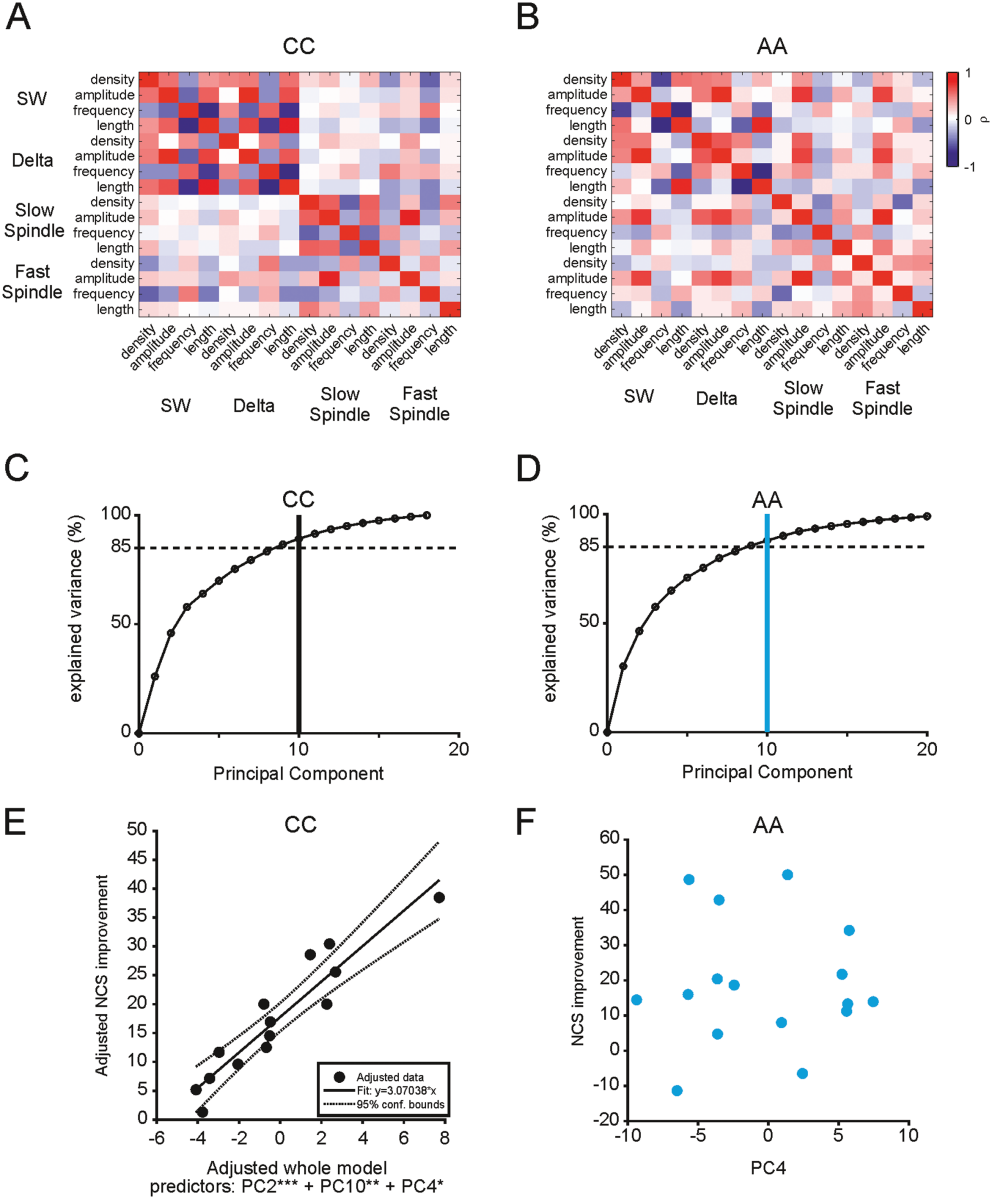
NREM event properties predict motor memory consolidation only in CC. (A) Matrix of correlation coefficients (ρ) for all N3 sleep event properties averaged across all electrodes for the CC carriers. (B) Matrix correlation coefficients (ρ) for all N3 sleep event properties averaged across all electrodes for the AA carriers. (C) Cumulative sum of explained variance per principal component for the PCA of N3 sleep variables in the CC group. (D) Cumulative sum of explained variance per principal component for the PCA of N3 sleep variables in the AA group. (E) Added variable plot for the final multilinear regression model using N3 sleep variable principal components 1−10 from CC carriers. *F*-test *p*-values for individual components added to the final model: *** *p* < 0.001, ** *p* < 0.01, * *p* < 0.05, n.s.: not significant. (F) In AA carriers a scatter plot of PC4 (shortest Euclidean distance to PC2 in CC) against improvement in NCS shows no relationship.

**Figure 7.**
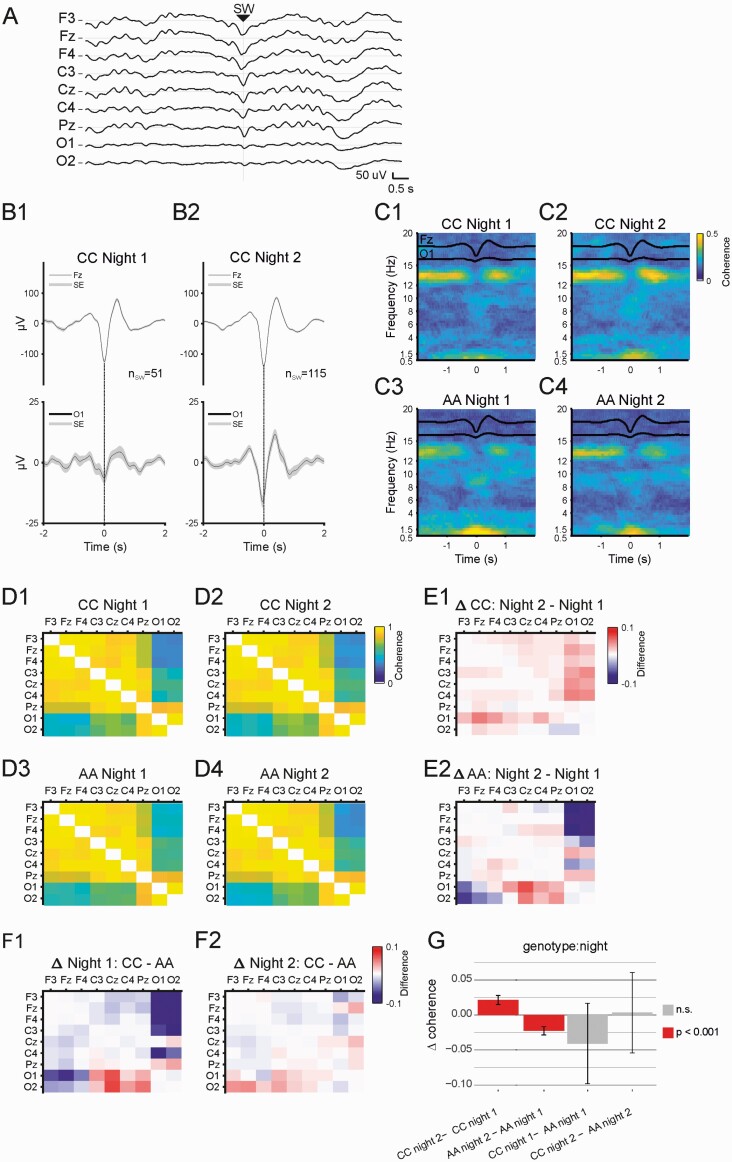
Slow wave coherence. (A) Raw data example showing all EEG traces surrounding a typical SW event at Fz. (B) SW wave-triggered averages from one participant from band pass filtered (0.5−4 Hz) and EEG traces. Individual SW event traces were averaged in windows of ±2 seconds with the SW trough time set *t* = 0. B1) SW triggered average at Fz and O1 for 51 SW events detected during night 1 N2 sleep in one participant (CC, Night 1), B2) SW triggered averages as described in C1 using, 115 SW events during night 2 from the same participant (‘CC’, Night 2). (C) Average SW triggered coherograms. SW triggered data windows (SW trough time at Fz, *t* = 0) from the seed electrode (Fz) and target electrode (O1) were used to calculate multitaper coherograms for each data window pair. Coherograms were averaged for each participant and then averaged for each group and recording night. In the coherogram, lighter colors indicate higher coherence. Overlaid black traces are SW wave triggered averages for seed and target electrode (Fz, top and O1, bottom). C1) Average coherogram for CC night 1, C2) Average coherogram for CC night 2, C3) Average coherogram for AA night 1, C4) Average coherogram for AA night 2. (D) Average SW-triggered coherence values (0.5−1.5 Hz, −0.5−0.5 s) for all electrode pairs for each genotype group during N2 sleep for both recording nights. (D1) CC, during night 1; (D2) CC, during night 2, (D3) AA, during night 1, D4 AA, during night 2. (E) Differences in SW coherence between nights in each genotype group CC (E1), and AA (E2). (F) Differences between genotype groups on each night, night 1: CC-AA (F1), night 2: CC-AA (F2). (G) Estimated marginal means differences for the factors genotype and night estimated from a linear mixed model analysis of SW event coherence. CC individuals show an increase in SW coherence during night 2, but AA’s show an overall decrease in coherence. Error bars indicate 95% confidence intervals.

**Figure 8.**
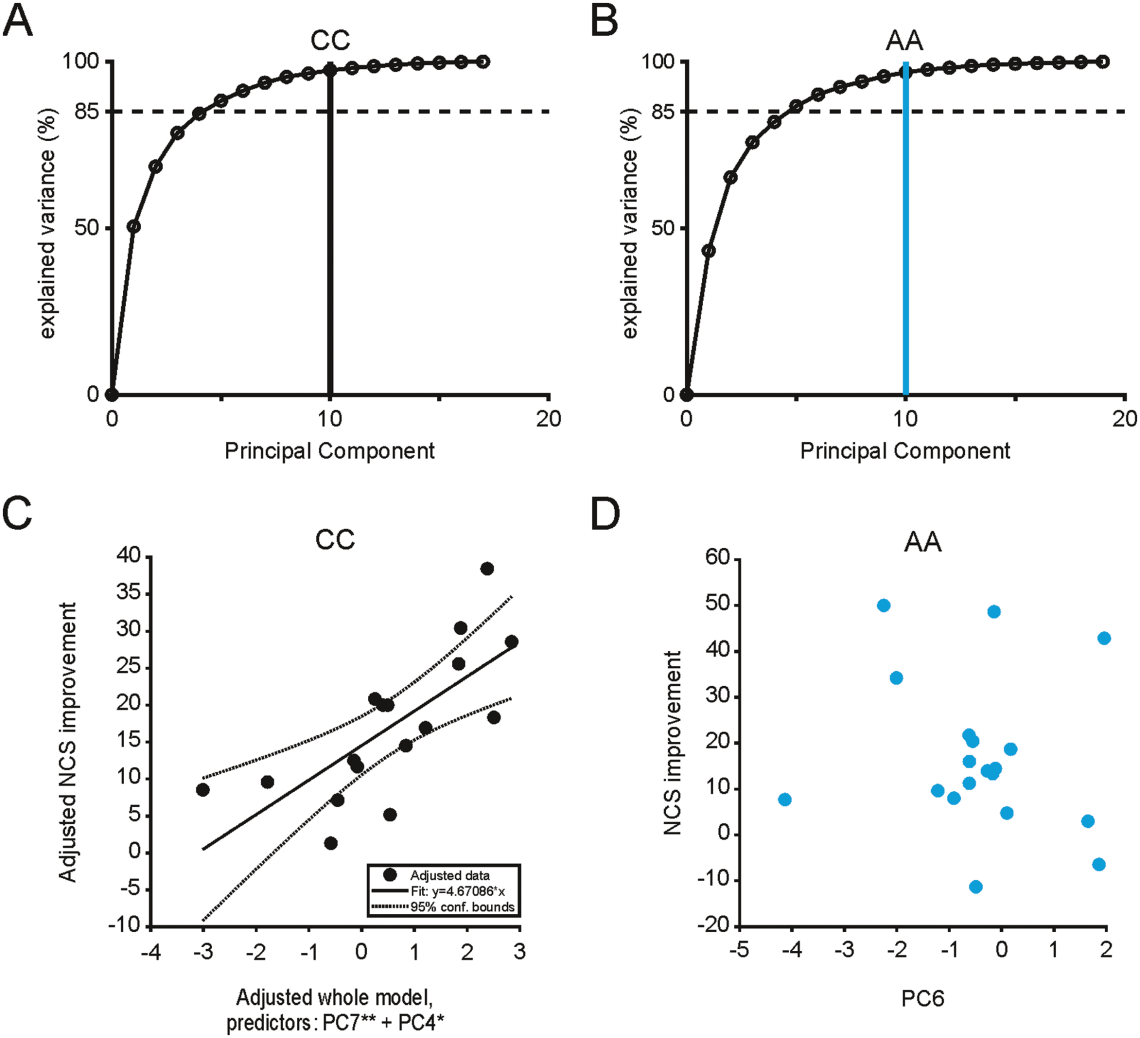
Slow wave coherence predicts motor memory consolidation only in CC. (A) Cumulative sum of explained variance per principal component for the PCA of N3 SW coherence in the CC group. (B) Cumulative sum of explained variance per principal component for the PCA of N3 SW coherence in the AA group. (C) Added variable plot for the final multilinear regression model using N3 SW coherence principal components 1–10 as predictors of overnight improvement in NCS in CC carriers. *F*-test *p*-values for individual components added to the final model: ** *p* < 0.01, * *p* < 0.05. D). In AA carriers a scatter plot of PC6 (shortest Euclidean distance to PC7 in CC) against improvement in NCS shows no relationship.

We did not detect differences in density of SW events between nights or genotypes (not shown). However, a linear mixed model analysis of SW amplitudes from N2 and N3 sleep from all electrode locations suggested a main effect of night and an important interaction term (night by genotype) in the initial full model. After stepwise reduction, the night by genotype interaction remained (*F*(1, 1356.05) = 18.67, *p* = 1.67e−05). Figure 4, panels C1−C4 show topographic plots of SW event amplitude, averaged for both genotype groups and recording nights. Figure 4, D shows the differences in estimated marginal means between nights, demonstrating an increase in SW amplitudes from night 1 (baseline) to night 2 (learning) in CC participants (night 1: 100 ± 4.80 μV; night 2: 105.12 ± 4.78 μV, *p* < 0.001) but not in AA (night 1: 112.2891 ± 4.34 μV, night 2: 111 ± 4.34 μV, n.s.). The SW event results were supported by similar results for delta wave (1.5-4 Hz) event properties. All SW and delta event properties are reported as Supplementary Tables S3–S6.

Collectively, these analyses suggest that the coordinated firing of cortical populations during SW events may be modulated following learning in a genotype-dependent manner.

### Spindle properties depend on experience, with differential effects of genotype

To further assess neurophysiological signatures of NREM sleep after motor learning we next extracted slow (9−12 Hz) and fast spindle (13−16 Hz) events. All slow and fast spindle properties are reported in Supplementary Tables S7–S10.

Figure 5, A shows a fast spindle triggered average trace at electrode Cz during N2 sleep, for both genotype groups and recording night, indicating that overall spindle morphology is comparable between genotype groups.

A linear mixed model analysis of slow spindle event properties revealed night and genotype dependent associations with amplitude, with a trend for an increase after learning in the CC group (31.1 ± 2.0 µV during night 1 vs. 31.7 ± 2.0 µV during night 2, *p* = 0.05), but a decrease in the AA group (from 33.8 ± 1.8 to 33.2 ± 1.8 µV, *p* = 0.03). We also observed a main effect for night-dependent associations with slow spindle frequency, with small decreases in slow spindle frequency after motor learning in both groups (from 11.36 ± 0.05 to 11.31 ± 0.05 Hz in CC, and from 11.33 ± 0.04 to 11.31 ± 0.04 Hz in AA, *p* = 0.005).

Next, we analyzed fast spindle event properties. Figure 5, B1–4 shows topographic plots of fast spindle amplitude at electrode Cz during N2 sleep for both genotype groups and recording nights. We detected a night by genotype interaction for fast spindle amplitude (*F*(1, 1356.05) = 10.24, *p* = 0.001), with differences in estimated marginal means shown in Figure 5, C: amplitudes increased from night 1 to night 2, but again only in the CC group participants (from 31.8 ± 2.0 to 32.6 ± 2.0 µV, *p* = 0.007). Fast spindle frequency did not vary across nights or genotype, but fast spindle duration showed a similar pattern to fast spindle amplitude: a genotype by night interaction (*F*(1, 1356.53) = 7.24, *p* = 0.0072), driven by shorter spindles in CC group during night 1 (795 ± 8 ms vs. 819 ± 8 ms in AA, *p* = 0.02) and an increase in spindle length from night 1 to night 2 only in the CC group (from 795 ± 8 to 800 ± 8 ms, *p* = 0.03). We found no strong evidence for an effect of genotype or night on slow or fast spindle density.

To summarize these NREM EEG event analyses, only the CC genotype group showed SW and spindle properties—particularly event amplitudes—that were sensitive to experience, sustaining increases on night 2 (post-MST learning) relative to night 1 (baseline). It is possible, then, that attenuated experience-dependent changes in thalamocortical activity contributed to more variable MST performance in the AA group. To better understand the relationship between night 2 sleep features and motor task performance improvement, we therefore performed regression analysis using NREM event properties recorded during night 2.

### NREM event properties predict motor memory consolidation only in CC carriers

To investigate whether NREM event properties during night 2 may predict overnight memory consolidation of motor learning, we first computed the Pearson’s linear correlation coefficients between NREM event properties averaged over all electrodes for each genotype group during night 2 (Figure 6). The correlation structure appeared qualitatively different between CC and AA, with a clear segmentation between covariance among the properties of low frequency events (SW and delta) and spindle properties evident in CC, but a more intermixed array of covariance in the AA participants. Indeed, Box’s *M*-test provided evidence for a difference between the covariance matrices of N3 sleep variables for CC and AA averaged over all electrodes (chi-square = 182.0666, *df* = 136, *p* = 0.0051).

Figure 6, A and B confirms that—as expected given the physiological inter-dependencies between SWs and spindles—NREM sleep event features tend to be highly correlated with one another, limiting the utility of multilinear regression of all the raw variables against behavior. We therefore performed PCA on NREM variables to derive independent data features for subsequent regression against MST performance. PCA behaved similarly for both genotype groups, with the first 10 principal components explaining over 85% of variance (Figure 6, C and D). We therefore entered these 10 principal components into a stepwise linear regression procedure to identify components that may predict sleep dependent memory consolidation. We then built multilinear regression models with a stepwise procedure, where terms were entered based on the squared sum of errors (SSE) for the final model. Principal components were included if, after an *F*-test, their inclusion improved the model at *p* < 0.01.

We built separate models for CC and AA genotype groups. For both N2 and N3 sleep variables regressed onto the MST performance measures of NCS or ET, stepwise multilinear regression successfully converged onto final models for the CC group—but did not include any terms for the AA group (Table 5). For example, for N3 sleep derived variables, multilinear regression identified a robust linear model predicting overnight improvements in the CC group’s MST performance on the basis of principal components 2, 4 and 10 (*F*(1,10) = 25.02, *p* =5.77e−05); Figure 6, E shows an adjusted variable plot for the final model. For comparison, Figure 6, F shows a scatter plot of AA PC4 (smallest Euclidean distance to PC2 in CC, not shown) against NCS improvement, which fails to show any linear relationship.

**Table 5.** Stepwise linear regression models of NREM event principal components

NREM stage	Group	MST variable	PC included	*F*	*p*-value
‘N2’	CC	NCS	PC4	6.63	0.024
‘N2’	AA	NCS		n.s.	n.s.
‘N2’	CC	ET	PC4	6.49	0.026
‘N2’	AA	ET		n.s.	n.s.
‘N3’	CC	NCS	PC2, PC4, PC10	25.02	5.77e-05
‘N3’	AA	NCS		n.s.	n.s.
‘N3’	CC	ET	PC2, PC7, PC10	21.63	0.0001
‘N3’	AA	ET		n.s.	n.s.

Consistent with the AA group’s more variable MST performance and limited post-training changes in SW and spindle event properties, these regression analyses confirm that network activity during NREM sleep can accurately predict behavior (i.e. overnight memory consolidation), but only in CC participants. However, since recent work has highlighted the importance of temporal interrelationships between thalamocortical oscillations for sleep-dependent memory consolidation [43, 47, 72, 73], we next tested whether SW coordination across the EEG recording locations also varied across nights and participants.

### Slow wave mediated cortical connectivity during NREM sleep

SWs can occur simultaneously at different locations across the cortex. Figure 7, A shows raw EEG traces surrounding a single SW event detected at electrode Fz. To illustrate SW-associated temporal covariance in frontal and occipital EEG, we used Fz SW events (trough times) as triggers to extract ±2 s windows of EEG surrounding each event across both channels, averaging across all windows for each recording night. Figure 7, B shows Fz SW event triggered averages at Fz and O1 from one participant of the CC group. Here highly stereotypical SW events are detected at Fz (with low variance) during both recording nights, but different average waveforms at O1. During night 1, Fz SW coincided with highly variable activity at O1, where a SW-like waveform is hardly separated from surrounding background activity (Figure 7, B1); in contrast, during night 2, a distinct average SW waveform coordinated with Fz manifests at O1 (Figure 7, B2).

We quantified SW synchronization during NREM sleep for both genotype groups and nights using multi-taper spectral coherence. Figure 7, panels C1−4 show group-averaged coherograms for the electrode pair Fz-O1 for both recording nights and genotype groups: the most coherent frequency ranges are 0.5−1.5 Hz (SW) and fast spindle coherence (12−15 Hz). We used the average SW coherence (0.5−1.5Hz) during 1s windows surrounding each SW for each electrode pair to construct a cortex-wide SW connectivity matrix. Figure 7, D1−4 show matrices of group averaged coherence values for both genotypes and both recording nights during N3 sleep. All matrices show a gradient of coherence, with highest values between frontal and central electrodes and lowest values between the most distant pairs, that is frontal and occipital electrodes.

We calculated the difference between all coherence values and plotted them in the same matrix layout to illustrate changes in SW coherence between nights (Figure 7, E1−2) and genotypes (Figure 7, F1−2). Consistent differences in SW coherence are apparent between recording nights. SW coherence increases in the CC group from night 1 (baseline) to night 2 (learning, Figure 7, F1)—but a decrease can be seen in the difference matrices for the AA genotype group (Figure 7, F2). A linear mixed model with subsequent stepwise reduction (Supplementary Table S11, Figure 7, G) revealed a genotype by night interaction, indicating a differential effect of learning on SW coherence in CC vs. AA genotypes (genotype × night: *F*(1, 11182) = 97.37, *p* < 2e−16). Both genotypes show changes in SW coherence upon motor learning, but a least squares estimation of group marginal means reveals that those in the CC group show a post-learning increase in SW coherence (CC night 1 0.85 ± 0.021, CC night 2 0.87 ± 0.019, *p* < 0.001, Supplementary Table S12), whereas the AA group show a decrease in overall SW coherence after learning (genotype by night: AA night 1 0.89 ± 0.019 − AA night 2 0.86 ± 0.021, *p* < 0.001).

Thus, the CC group showed the increased SW coherence predicted by previous studies [43, 47], whereas SW coordination was attenuated following learning in the AA participants.

### Slow wave coherence predicts motor memory consolidation only in CC carriers

To further elucidate the relationship between SW-associated connectivity and overnight MST performance changes we performed PCA combined with multiple linear regression analysis, as described for NREM sleep event features in Figure 6. Figure 8 A and B shows explained variance plots for PCA in both groups for N3 SW coherence.

N2 and N3 sleep SW coherence regression models for MST performance (NCS or ET), converged onto final models for the CC group – but, as for individual NREM events in Figure 6, did not include any terms for the AA group (Table 6). For example, for N3 sleep derived variables, multilinear regression identifies a linear model with PC 2, 4 and 10 (*F*(1,10) = 10.03, *p* = 0.0008); Figure 8, C shows an adjusted variable plot for the final model for N3 SW coherence principal components regressed onto the improvement in NCS. Figure 8, D shows a scatter plot of AA PC4 (smallest Euclidean distance to PC2 in CC, not shown) against NCS improvement.

**Table 6.** Stepwise linear regression of SW coherence principal components

NREM stage	Group	MST variable	PC included	*F* stat	*p*-value
‘N2’	CC	NCS		n.s.	n.s.
‘N2’	AA	NCS		n.s.	n.s.
‘N2’	CC	ET	PC4	7.97	0.013
‘N2’	AA	ET		n.s.	n.s.
‘N3’	CC	NCS	PC4, PC7	7.98	0.005
‘N3’	AA	NCS		n.s.	n.s.
‘N3’	CC	ET	PC2, PC4, PC6, PC7	10.13	0.0008
‘N3’	AA	ET		n.s.	n.s.

In a similar pattern to the analysis of NREM event features, SW coherence only predicts overnight memory consolidation in CC carriers but not in AA carriers. This again indicates the rs1344706 genotype-dependence of NREM neurophysiology’s utility as a predictor of sleep-dependent behavior.

## Discussion

We performed a recall-by-genotype study [46] to investigate the potential contributions of an SZ-associated SNP, rs1344706, to sleep-dependent memory processing and neurophysiology in healthy volunteers. In summary, (1) all participants showed normal wake/sleep rhythms and sleep architecture; (2) we observed greater variance in learning and sleep-dependent memory consolidation following a motor task in AA participants; (3) we detected genotype- and learning-dependent effects on SW and fast spindle amplitudes, with the AA group showing normal SW and spindle densities, but attenuated changes in SW and spindle amplitudes after learning; (4) the AA group also failed to exhibit the learning-dependent increase in SW coherence evident in the CC genotype group; (5) consequently, metrics of coordinated network activity during NREM sleep were robust predictors of sleep-dependent memory consolidation in CC participants, but were unable to predict behavior in the AA group associated with higher genetic liability for schizophrenia.

### Motor memory consolidation

Using an MST, performance in which has previously shown to be impaired in patients with schizophrenia, we found evidence for greater variability in overnight improvement and other variables derived from MST in those with the AA genotype at rs1344706, suggesting that rs1344706 may associate with subtle changes in motor learning and consolidation.

Motor learning [74] and its sleep-dependent memory consolidation are impaired in patients with schizophrenia [53, 75–77] and the key brain areas involved in these traits, including the neocortex, striatum, thalamus, hippocampus and cerebellum [78–81], have all been implicated in the etiology of SZ [82–84]. *ZNF804A* has been shown to be highly expressed in these brain regions, particularly the thalamus, hippocampus and cortex [85], hence altered *ZNF804A* function or expression may contribute to changes in brain development and plasticity that influence motor learning and its consolidation [86]. Previous studies have shown that variability between individuals during early phases of learning a motor task is higher in patients diagnosed with SZ compared to healthy controls [87], potentially reflecting higher variability in brain anatomy or functional connectivity patterns [88]. Whether this variability and the associations of *ZNF804A* derive from neurodevelopmental effects or altered adult neural plasticity remains an open question.

### NREM sleep neurophysiology

Detailed analyses of overnight EEG unveiled relationships between rs1344706, corticothalamic activity during NREM sleep and neural correlates of motor memory consolidation. We observed several interaction effects between genotype and recording night, where NREM sleep activity appears to be differentially affected by the acquisition of a motor task in AA as compared to CC participants.

*Spindle oscillations*. Previous studies have shown that sleep-dependent motor memory consolidation correlates with spindle oscillations [62, 72, 89–91]. Indeed, a substantial body of work has demonstrated correlations between N2 sleep or spindles with motor memory in healthy participants [92–95], although contradictory studies do exist [96, 97]. In particular, the individual contributions of slow and fast spindles to memory consolidation are still debated, though their dissociable topographies may reflect distinct roles in processing different stages or types of memory consolidation.

We found some evidence supporting a role for slow spindle oscillations (9−12 Hz) in motor memory consolidation, since slow spindle amplitudes appeared to be increased in CC genotype participants during the learning night, whilst decreasing in the AA group; fast spindle amplitudes and durations also increased after learning, again only in the CC genotype group. The mechanisms driving these experience-dependent changes remain unknown, but may relate to plasticity in cortico-thalamic feedback, which has been shown to modulate spindle initiation and termination [98].

We found no evidence of an effect of rs1344706 genotype or night on either slow or fast spindle densities. Previous studies demonstrated reduced fast spindle density or integrated spindle activity in first episode [34, 99] and chronically ill patients [32, 33, 39], plus in their first-degree relatives [34, 44, 100]. Meanwhile, reduced fast spindle density has been reported in healthy carriers of a catechol-O-methyltransferase (COMT) polymorphism [101], while polygenic risk score for SZ [102] was positively correlated with higher fast spindle density and amplitudes in healthy adolescents. The situation therefore remains complex and, given established polygenic effects in SZ, multiple genetic variants and their interactions are likely to impact cortico-thalamic circuit development and activity in different ways. In particular, SNPs linked to ion channel genes like *CACNA1C* [102] may interact with other SNPs to impact corticothalamic development and maturation which might have causal effects on cortico-thalamic oscillatory signatures or NREM sleep [40].

*Slow oscillations*. On average, SW amplitudes increased during the sleep after learning only in the CC genotype group. In addition, SW coherence appears to be differentially modulated after learning between the genotype groups: those in the CC group show an increase in SW coherence, but participants with the AA genotype show a decrease in SW coherence during the night that followed motor learning. Previous studies have shown that during early sleep, after motor learning, SW event amplitudes are locally increased in central and parietal areas [103]. SW coherence has also been shown to increase during sleep after a declarative memory task [47], and our recent work has demonstrated SW coherence increased after motor learning in a control group, but not in patients diagnosed with SZ [43]. D’Agostino et al. [44] report decreased SW amplitude and slope in first-degree relatives of schizophrenia patients, potentially indicating altered synaptic connectivity or plasticity in cortical networks. Our results in individuals homozygous for the ‘A’ allele at rs1344706 seem to be line with these findings and provide a genetic correlate for SW phenotypes related to psychosis and SZ.

### Correlation between NREM neurophysiology and motor memory consolidation

We observed a striking lack of predictability of motor memory consolidation in carriers of the risk variant AA - compared to the non-risk variant CC where both local (SW and spindle event properties) and distributed (SW coherence across the scalp) NREM sleep features predicted successful motor memory consolidation.

This lack of predictability of overnight memory consolidation in AA carriers is surprising and unexpected. A higher variability in behavioral responses and an altered correlational structure between sleep variables may destroy previously described associations between sleep neurophysiology and memory consolidation. Given the relatively high frequency of the ‘A’ allele in the general population (based on allele frequencies reported for the European arm of the 1000Genomes project [104] approximately 39% of the population are AA at this locus), these findings may explain some of the inconsistencies found in the literature that describe relationships between sleep neurophysiology and overnight improvement in motor learning.

Recent studies on the rodent homologue of *ZNF804A* suggest the gene has a role both during development and in adult plasticity [86, 105]. Our own work in a rodent neurodevelopmental model of SZ has demonstrated that interference in cortico-thalamic development causes severe disruption of SW coordination between remote cortical areas and simultaneous desynchronization of spindle and hippocampal ripple oscillations [59]. Given the suggested role of *ZNF804A* in cortical and thalamic development we speculate that rs1344706 may have a role in corticothalamic development which itself would be related to impaired coordination of SW activity during sleep. These deep characterizations of genotypic association motivate future mechanistic studies in animal models that enable high-resolution phenotyping of corticothalamic circuit development and plasticity, and their role in sleep dependent memory processing.

## Limitations and Conclusions

The functions of *ZNF804A* are not fully documented, nor are the molecular mechanisms linked to rs1344706 [13]. Indeed, the effects of rs1344706 may depend on other SNPs [106, 107] and environmental factors [108]. Also, the relatively small sample size of this study naturally limits the strength of our conclusions; future sleep studies deploying wearable technology to monitor sleep neurophysiology over extended periods of time and in much larger genotyped samples stand poised to generate powerful advances in this regard. It will also be important to extend studies of sleep’s genotype-associated contributions to other memory types, including declarative memories, since NREM oscillations have been associated with a range of memory impairments in schizophrenia [75, 109].

Given the complex network of events linking genetics to brain-wide connectivity and function, how can we best map genomic information to a neurobiological understanding of SZ? Here we show that sleep neurophysiology presents a uniquely powerful opportunity to bridge different levels of analysis: relating genotype to sleep-dependent physiology and environmental factors such as learning, constitutes a rational, neurobiologically informed approach to delineating causal mechanisms of thalamocortical circuit dysfunction [45]. Future translational studies should investigate the influence of *ZNF804A* on SW and spindle properties and their coordination in genetic rodent models and patient populations to further elucidate genetic and circuit mechanisms of psychosis and their impacts on sleep, cognition, and novel therapies.

## Supplementary Material

zsab191_suppl_Supplementary_Tables

## Preprint Repositories

A previous version of the manuscript has been added to biorxiv: https://doi.org/10.1101/2020.05.01.072165
