# Supplementary material for: Schizophrenia-associated variation at ZNF804A correlates with altered experience-dependent dynamics of sleep slow waves and spindles in healthy young adults
Source: Sleep. 2021 Jul 30;44(12):zsab191. doi: 10.1093/sleep/zsab191 (PMC8664578; doi:10.1093/sleep/zsab191)
Supplement: zsab191_suppl_Supplementary_Tables [file zsab191_suppl_supplementary_tables.docx]

Schizophrenia-associated variation at *ZNF804A* correlates with altered experience-dependent dynamics of sleep slow waves and spindles in healthy young adults

Authors:

Ullrich Bartsch*^1,2,#^, Laura J Corbin*^3,4^, Charlotte Hellmich^1^, Michelle Taylor^3^_,_ Kayleigh E Easey^3,5^, Claire Durant^6^, Hugh M Marston^2$^, Nicholas J Timpson *^3,4^, Matthew W Jones*^1^

* These authors contributed equally.

Affiliations:

1) School of Physiology, Pharmacology & Neuroscience, University of Bristol, Bristol BS8 1TD, UK

2) Translational Neuroscience, Eli Lilly & Co Ltd UK, Erl Wood Manor, Windlesham, UK

3) MRC Integrative Epidemiology Unit at University of Bristol, Bristol, UK

4) Population Health Sciences, Bristol Medical School, University of Bristol, Bristol, UK

5) UK Centre for Tobacco and Alcohol Studies, School of Psychological Science, University of Bristol, Bristol, UK

6) Clinical Research and Imaging Centre (CRIC), University of Bristol, Bristol, UK

# current address: UK DRI Health Care & Technology at Imperial College London and the University of Surrey, Surrey Sleep Research Centre, University of Surrey, Clinical Research Building, Egerton Road, Guildford, Surrey, GU2 7XP

$ current address: Böhringer Ingelheim Pharma GmbH & Co. KG, Biberach, Germany

Corresponding authors:

[n.j.timpson@bristol.ac.uk](mailto:n.j.timpson@bristol.ac.uk) ; [matt.jones@bristol.ac.uk](mailto:matt.jones@bristol.ac.uk)

# Supplementary Tables

## Table S1 - Habitual sleep behavior and diurnal rhythms derived from actigraphy

|  | Mean (SD) | |  |
| --- | --- | --- | --- |
|  | CC group | AA group | p^a^ |
| Sleep analysis | N=16 | N=19 |  |
| # nights in analysis | 14.3 (1.8) | 14.3 (1.7) | 0.75 |
| TIB (minutes) | 498 (31) | 490 (35) | 0.49 |
| TST (minutes) | 403 (35) | 393 (39) | 0.39 |
| SOL (minutes) | 10 (7) | 11 (8) | 0.86 |
| Sleep efficiency (%) | 81 (5) | 80 (6) | 0.62 |
| FI | 28.7 (8.4) | 27.8 (6.1) | 0.82 |
| NPCRA analysis | N=15 | N=19 |  |
| # days in analysis | 13.3 (1.5) | 13.1 (1.9) | 0.79 |
| RA | 0.71 (0.08) | 0.73 (0.06) | 0.44 |
| IS | 0.68 (0.11) | 0.72 (0.12) | 0.21 |
| IV | 0.35 (0.07) | 0.34 (0.05) | 0.66 |
| L5 | 8.98 (2.51) | 8.12 (1.82) | 0.38 |
| M10 | 52.1 (4.0) | 52.5 (2.8) | 0.96 |

SD, standard deviation; TIB, time in bed; TST, total sleep time; SOL, sleep onset latency; FI, fragmentation index; RA, relative amplitude; IS, interdaily stability; IV, intra-daily variability; L5, least 5 average (L5); M10, most 10 average.

^a^ *p*-value from Wilcoxon rank-sum (Mann-Whitney) test

## Table S2 - PSG-derived sleep architecture across groups and sessions

|  | **Mean (SD)** | | | | **Mixed model output** | |
| --- | --- | --- | --- | --- | --- | --- |
|  | **CC group (N=18)** | | **AA group (N=22)** | | **Night effect** | **Genotype group effect** |
| **Outcome measure** | **Night 1** | **Night 2** | **Night 1** | **Night 2** | **Beta^a^ (SE),**  **F(df1,df2) , *p*** | **Beta^b^ (SE),**  **F(df1,df2) , *p*** |
| **TIB (minutes)** | 498 (44) | 505 (47) | 497 (41) | 497 (40) | b = 2.89 (5.39),  F = 0.29 (1, 39),  p = 0.60 | b = -4.41 (12.42),  F = 0.13 (1, 38),  p = 0.72 |
| **TST (minutes)** | 457 (46) | 463 (47) | 448 (56) | 462 (43) | b = 9.91 (6.22),  F = 2.54 (1, 39),  p = 0.12 | b = -5.40 (14.11),  F = 0.15 (1, 38)  p = 0.70 |
| **Stage N1 %** | 7.3 (3.2) | 6.6 (3.0) | 8.2 (2.7) | 6.8 (2.3) | b = -1.09 (0.35),  F = 9.47 (1, 39),  p = 3.8 x 10^-03^ | b = 0.50 (0.82),  F = 0.37 (1, 38),  p = 0.55 |
| **Stage N2 %** | 46.7 (7.1) | 42.2 (12.2) | 44.0 (7.7) | 44.2 (7.5) | b = -1.91 (1.27),  F = 2.25 (1, 39),  p = 0.14 | b = -0.37 (2.49),  F = 0.02 (1, 38)  p = 0.88 |
| **Stage N3 %** | 23.3 (6.3) | 23.5 (4.6) | 25.0 (6.1) | 25.4 (5.9) | b = 0.32 (0.51),  F = 0.41 (1, 39)’  p = 0.52 | b = 1.81 (1.76),  F = 1.05 (1, 38),  p = 0.31 |
| **REM %** | 22.7 (3.8) | 24.7 (5.6) | 22.7 (5.7) | 23.7 (4.9) | b = 1.43 (0.71),  F = 4.12 (1, 39),  p = 0.05 | b = -0.54 (1.45),  F = 0.14 (1, 38),  p = 0.71 |
| **SOL (minutes)** | 17 (12) | 14 (9) | 12 (7) | 10 (5) | b = -2.67 (1.53),  F = 3.06 (1, 39),  p = 0.09 | b = -3.97 (2.20),  F = 3.25 (1, 38),  p = 0.08 |
| **WASO (minutes)** | 24 (12) | 28 (22) | 37 (26) | 25 (18) | b = -4.35 (4.34),  F = 1.01 (1, 39),  p = 0.32 | b = 4.96 (4.89),  F = 1.03 (1, 38),  p = 0.32 |
| **Sleep efficiency (%)** | 92 (4) | 92 (4) | 90 (6) | 93 (4) | b = 1.58 (0.91),  F = 2.99 (1, 39),  p = 0.09 | b = -0.35 (1.20),  F = 0.09 (1, 38),  p = 0.77 |

SD, standard deviation; TIB, time in bed; TST, total sleep time; SOL, sleep onset latency; WASO, wake after sleep onset.

^a^ given with respect to night 1 as baseline; ^b^ given with respect to the CC group as baseline.

## Table S3 - Linear mixed model results for NREM SW event properties

|  | Stepwise reduced linear mixed model output | | | | | | | | |
| --- | --- | --- | --- | --- | --- | --- | --- | --- | --- |
|  | **Night effect** | | | **Genotype effect** | | | **Interaction (Night X Genotype)** | | |
|  | Beta (SE) | F(df1,df2) | p | Beta (SE) | F(df1,df2) | p | Beta (SE) | F(df1,df2) | p |
| **SW density**  **(N/min)** | 1.920e-02  (1.864e-02) | 1.0610  (1, 1373.18) | 0.3032 | 2.741e-02  (3.933e-02) | 0.4858  (1, 47.14) | 0.4892 | 8.772e-03  (2.515e-02) | 0.1216  (1, 1373.21) | 0.7273 |
| **SW amplitude**  **(μV)** | 4.5681  (0.9865) | 21.4405  (1, 1356.04) | 4.00e-06  ***^,B^ | 11.7354  (6.4694) | 0.8591  (1,38.81) | 0.0774 | -5.7392 (1.3284) | 18.6669  (1, 1356.05) | 1.67e-05  ***^,B^ |
| **SW frequency**  **(Hz)** | -1.633e-03  (2.807e-03) | 0.3387  (1, 1356.45) | 0.5607 | -9.669e-03  (6.739e-03) | 2.0586  (1, 44.77) | 0.1583 | -8.985e-05  (3.779e-03) | 0.0006  (1, 1356.41) | 0.9810 |
| **SW length**  **(duration, s)** | 9.012e-04  (2.425e-03) | 0.1381  (1, 1356.25) | 0.7102 | 3.105e-03  (7.616e-03) | 0.1662  (1, 41.76) | 0.6856 | -4.854e-04  (3.265e-03) | 0.0221  (1, 1356.23) | 0.8819 |

___ = full model output, no evidence of night or genotype effect

n.s. not significant i.e. term removed in final model, * p<0.05, ** p<0.01, ***p<0.001, ^B^ p < 0.003125 (Bonferroni corrected)

## Table S4 - Estimated marginal means of NREM SW event properties across all electrodes:

|  | **CC group (N=18)** | | **AA group (N=22)** | |
| --- | --- | --- | --- | --- |
|  | Night 1 | Night 2 | Night 1 | Night 2 |
| **SW density**  **(N/min)** | 0.6117  (0.0292) | 0.6309  (0.0291) | 0.6391  (0.0264) | 0.6671  (0.0264) |
| **SW amplitude**  **(μV)** | 100.5537  (4.7984) | 105.1218  (4.7975)*** | 112.2891  (4.3392) | 111.118  (4.34) |
| **SW frequency**  **(Hz)** | 1.1707  (0.005) | 1.1691  (0.005) | 1.1611  (0.0045) | 1.1593  (0.0045) |
| **SW length**  **(duration, s)** | 0.7821  (0.0057) | 0.783  (0.0056) | 0.7852  (0.0051) | 0.7857  (0.0051) |

Mean (SE), within genotype, between night comparisons: # p<0.1, * p<0.05, ** p<0.01, ***p<0.001

## Table S5 - Linear mixed model results for NREM delta wave event properties

|  | Stepwise reduced linear mixed model output | | | | | | | | |
| --- | --- | --- | --- | --- | --- | --- | --- | --- | --- |
|  | **Night effect** | | | **Genotype effect** | | | **Interaction (Night X Genotype)** | | |
|  | Beta (SE), | F(df1,df2), | p | Beta (SE), | F(df1,df2), | p | Beta (SE), | F(df1,df2), | p |
| **Delta density**  **(N/min)** | 5.019e-02  (3.007e-02) | 2.7872  (1, 1373.12) | 0.09525 | 4.796e-02  (7.697e-02) | 0.3883  (1, 43.88) | 0.53645 | -5.819e-02  (4.057e-02) | 2.0571  (1, 1373.14) | 0.15172 |
| **Delta amplitude**  **(μV)** | 2.9055  (0.8777) | 10.9584  (1 1364.03) | 0.000956  ***^,B^ | 8.7948  (5.4417) | 2.9055  (1 38.92) | 0.1141 | -3.6481  (1.1837) | 9.4984  (1, 1364.03) | 0.0020977  **^,B^ |
| **Delta frequency**  **(Hz)** | 0.009 (0.0035) | 6.5674  (1,1365) | 0.01049  * | n.s. | n.s. | n.s. | n.s. | n.s. | n.s. |
| **Delta length**  **(duration, s)** | 2.852e-03  (2.269e-03) | 1.5800  (1, 1364.13) | 0.2090 | 3.285e-03  (7.126e-03) | 0.2126  (1, 41.77) | 0.6472 | -1.403e-03  (3.060e-03) | 0.2103  (1, 1364.13) | 0.6466 |

___ = full model output, no evidence of night or genotype effect,

n.s. not significant i.e. term removed in final model, * p<0.05, ** p<0.01, ***p<0.001, ^B^ p < 0.003125 (Bonferroni corrected)

## Table S6 - Estimated marginal means of NREM delta wave event properties across all electrodes

|  | **CC group (N=18)** | | **AA group (N=22)** | |
| --- | --- | --- | --- | --- |
|  | Night 1 | Night 2 | Night 1 | Night 2 |
| **Delta density**  **(N/min)** | 1.1007  (0.0571) | 1.1509  (0.0571) | 1.1487  (0.0516) | 1.1407  (0.0516) |
| **Delta amplitude (μV)** | 92.1743  (4.0357) | 95.0798  (4.0355)*** | 100.9691  (3.6504) | 100.2266  (3.6504) |
| **Delta frequency (Hz)** | 2.2061  (0.0114) | 2.1978  (0.0114) | 2.1942^+^  (0.0103) | 2.1846^+^  (0.0103) |
| **Delta length**  **(duration, s)** | 0.5571  (0.0053) | 0.5599  (0.0053) | 0.5604  (0.0048) | 0.5618  (0.0048) |

Mean (SE), within genotype, between night comparisons: # p<0.1, * p<0.05, ** p<0.01, ***p<0.001

## Table S7 - Linear mixed model results for NREM slow spindle event properties

|  | Stepwise reduced linear mixed model output | | | | | | | | |
| --- | --- | --- | --- | --- | --- | --- | --- | --- | --- |
|  | **Night effect** | | | **Genotype effect** | | | **Interaction (Night X Genotype)** | | |
|  | Beta (SE) | F(df1,df2) | p | Beta (SE) | F(df1,df2) | p | Beta (SE) | F(df1,df2) | p |
| **Slow spindle**  **density**  **(N/min)** | 0.03277  (0.03489) | 0.8824  (1, 1357.31) | 0.56 | 0.06791  (0.11473) | 0.3503  (1, 41.42) | 0.3477104 | -0.07445  (0.04712) | 2.4957  (1, 1357.36) | 0.1143902 |
| **Slow spindle**  **amplitude (μV)** | 0.6354  (0.3302) | 4.8377  (1, 1331.06) | 0.054524  # | 2.7344  (2.7174) | 1.0125  (1, 38.52) | 0.320585 | -1.2918  (0.4451) | 8.4238  (1, 1331.07) | 0.003764  ** |
| **Slow spindle**  **frequency (Hz)** | -4.674e-02  (1.646e-02) | 9.2693  (1,1332.3) | 0.0024  ** | n.s. | n.s. | n.s. | n.s. | n.s. | n.s. |
| **Slow spindle**  **length**  **(duration, s)** | 0.010  (0.0065) | 3.3330  (1,1331.81) | 0.068 | 1.591e-03  (2.251e-02) | 0.0050  (1, 45.30) | 0.94396 | 1.262e-02  (1.300e-02) | 0.9418  (1 1331.93) | 0.33200 |

___ = full model output, no evidence of night or genotype effect

n.s. not significant i.e. term removed in final model, * p<0.05, ** p<0.01, ***p<0.001, ^B^ p < 0.003125 (Bonferroni corrected)

## Table S8 - Estimated marginal means of NREM slow spindle event properties across all electrodes

|  | **CC group (N=18)** | | **AA group (N=22)** | |
| --- | --- | --- | --- | --- |
|  | Night 1 | Night 2 | Night 1 | Night 2 |
| **Slow spindle density**  **(N/min)** | 0.5531  (0.0851) | 0.5858  (0.085) | 0.621  (0.077) | 0.5793  (0.077) |
| **Slow spindle amplitude (μV)** | 31.0691  (2.0154) | 31.7045  (2.0151) ^#^ | 33.8034  (1.8228) | 33.1471  (1.8229) * |
| **Slow spindle frequency (Hz)** | 11.3617  (0.0469) | 11.315  (0.0469) | 11.3283  (0.0424) | 11.3054  (0.0424) |
| **Slow spindle length**  **(duration, s)** | 0.8749  (0.0167) | 0.8573  (0.0167) | 0.8733  (0.0151) | 0.8684  (0.0151) |

Mean (SE), within genotype, between night comparisons: ^#^ p<0.1, * p<0.05, ** p<0.01, ***p<0.001

## Table S9 - Linear mixed model results for NREM fast spindle event properties

|  | Stepwise reduced linear mixed model output | | | | | | | | |
| --- | --- | --- | --- | --- | --- | --- | --- | --- | --- |
|  | **Night effect** | | | **Genotype effect** | | | **Interaction (Night X Genotype)** | | |
|  | Beta (SE) | F(df1,df2) | p | Beta (SE) | F(df1,df2) | p | Beta (SE) | F(df1,df2) | p |
| **Fast spindle density**  **(N/min)** | -4.142e-02  (4.174e-02) | 0.9844  (1, 1357.21) | 0.3213 | -1.264e-01  (1.660e-01) | 0.5797  (1, 40.29) | 0.4508 | 1.102e-02  (5.639e-02) | 0.0382  (1, 1357.24) | 0.8450 |
| **Fast spindle amplitude (μV)** | 0.8243 (0.3061) | 7.2541 (1, 1356.04) | 0.0072  ** | 2.3894  (2.6588) | 0.8076  (1, 38.46) | 0.3744 | -1.3232  (0.4136) | 10.2360  (1, 1356.05) | 0.0014  **^,B^ |
| **Fast spindle frequency (Hz)** | 3.897e-03  (1.145e-02) | 0.1157  (1, 1356.07) | 0.7338 | -9.165e-03  (8.061e-02) | 0.0129  (1, 38.71) | 0.9101 | -2.051e-02  (1.548e-02) | 1.7565  (1, 1356.08) | 0.1853 |
| **Fast spindle length**  **(duration, s)** | 8.353e-03  (3.750e-03) | 4.9621  (1, 1356.42) | 0.026073  * | 2.466e-02  (1.060e-02) | 1.0824  (1, 42.73) | 0.02607  * | -1.363e-02  (5.067e-03) | 7.2399  (1, 1356.53) | 0.0072  ** |

___ = full model output, no evidence of night or genotype effect

n.s. not significant i.e. term removed in final model, * p<0.05, ** p<0.01, ***p<0.001, ^B^ p < 0.003125 (Bonferroni corrected)

## Table S10 - Estimated marginal means of NREM fast spindle event properties across all electrodes

|  | **CC group (N=18)** | | **AA group (N=22)** | |
| --- | --- | --- | --- | --- |
|  | Night 1 | Night 2 | Night 1 | Night 2 |
| **Fast spindle density**  **(N/min)** | 1.7274  (0.1231) | 1.6859  (0.1231 | 1.601  (0.1114) | 1.5706  (0.1114) |
| **Fast spindle amplitude (μV)** | 31.8086  (1.9719) | 32.6329  (1.9717)* | 34.198  (1.7836) | 33.6991  (1.7837) |
| **Fast spindle frequency (Hz)** | 12.8534  (0.0598) | 12.8573  (0.0598) | 12.8442  (0.0541) | 12.8276  (0.0541) |
| **Fast spindle length**  **(duration, s)** | 0.7946  (0.0079) | 0.8030  (0.0079)* | 0.8193  (0.0071)^‡^ | 0.8140  (0.0071) |

Mean (SE), within genotype, between night comparisons: # p<0.1, * p<0.05, ** p<0.01, ***p<0.001

Within night, between genotype effect: ‡ p<0.05

## Table S11 - Linear mixed model results for SW triggered slow coherence

|  | Mixed model output ^b^ | | | | | | | | |
| --- | --- | --- | --- | --- | --- | --- | --- | --- | --- |
|  | **Night effect** | | | **Genotype effect** | | | **Interaction (Night X Genotype)** | | |
|  | Beta^c^ (SE) | F(df1,df2) | p | Beta^c^ (SE), | F(df1,df2) | p | Beta^c^ (SE) | F(df1,df2) | p |
| **SW triggered**  **slow coherence**  **(0.5-1.5 Hz)** | -3.305e-03 (2.838e-02) | 0.0455  (1, 11182) | 0.5128 | -3.305e-03 (2.838e-02) | 0.4365  (1, 38) | 0.8310 | 4.399e-02 (4.458e-03) | 97.3702  (1, 11182) | <2e-16  *** |

## Table S12 - Estimated marginal means of SW triggered SW coherence across all electrodes

|  |  | | | |
| --- | --- | --- | --- | --- |
|  | **CC group (N=18)** | | **AA group (N=22)** | |
|  | Night 1 | Night 2 | Night 1 | Night 2 |
| **SW triggered**  **slow coherence**  **(0.5-1.5 Hz)** | 0.8498916  (0.021) | 0.8714120  (0.019)*** | 0.8905784  (0.019) | 0.8681068  (0.021)*** |

mean(atanh(coherence)) (SE), within genotype, between night comparisons: # p<0.1, * p<0.05, ** p<0.01, ***p<0.001
